# Supplementary material for: A rationally designed JAZ subtype-selective agonist of jasmonate perception
Source: Nat Commun. 2018 Sep 7;9:3654. doi: 10.1038/s41467-018-06135-y (PMC6128907; doi:10.1038/s41467-018-06135-y)
Supplement: Supplementary file 1 — Supplementary Information [file 41467_2018_6135_MOESM1_ESM.pdf]

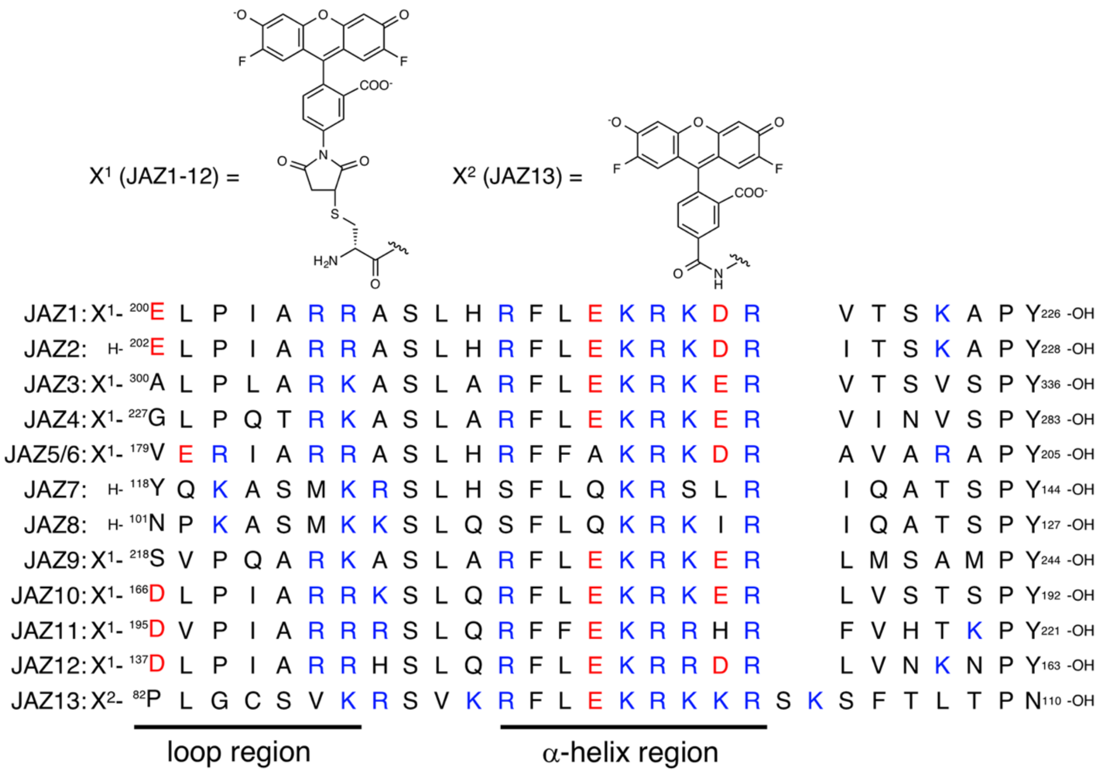

Supplementary Figure 1. OG-conjugated JAZ peptides.

Chemical structures of OG-derivatives as the epitope tags used in this study. Amino acid sequence alignment around the Jas motif of 13 *Arabidopsis thaliana* JAZs was coupled with X^1 (JAZ1-12) or X^2 (JAZ13). Acidic amino acids (D and E) are shown in red, and basic ones (K and R) are shown in blue.

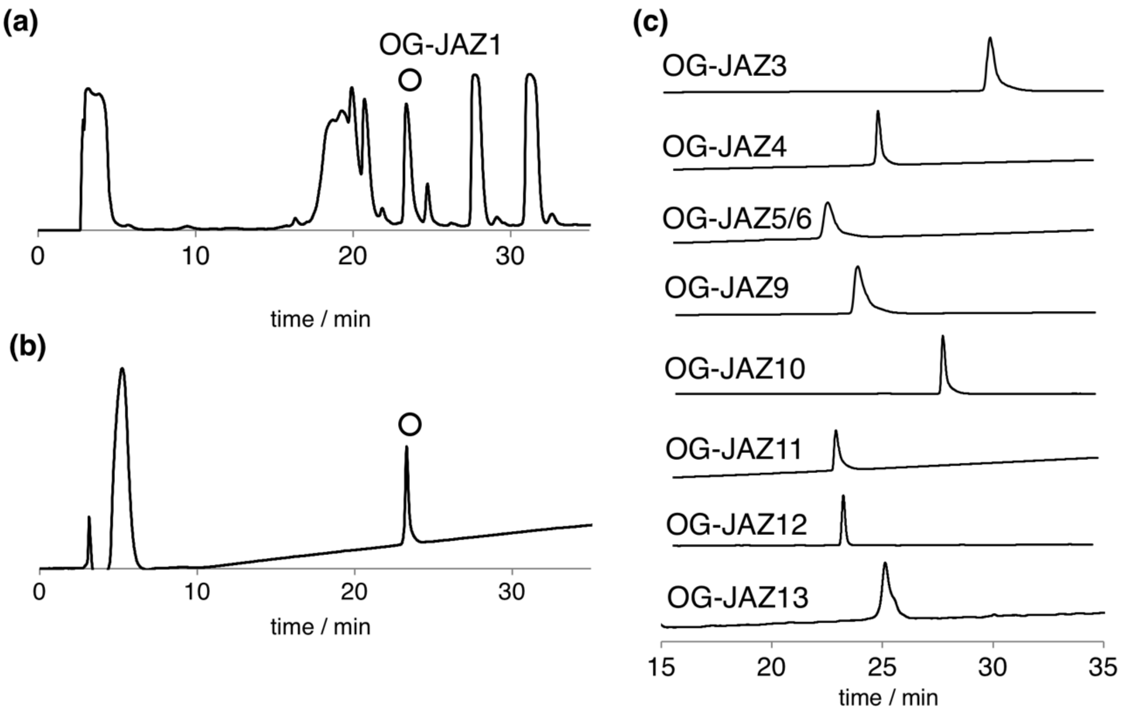

Supplementary Figure 2. Purification of OG-conjugated JAZ peptides.

(**a, b**) HPLC charts of (**a**) the reaction mixture or (**b**) purified OG-conjugated JAZ1 peptide. (**c**) HPLC charts of purified OG-conjugated JAZ peptide (3–5/6 and 9–13). Linear gradient conditions are shown in the synthetic procedure as follows: A (CH_3CN with 0.05% TFA): B (H_2O with 0.05% TFA) = 20:80 (5 min) to 40:60 (35 min).

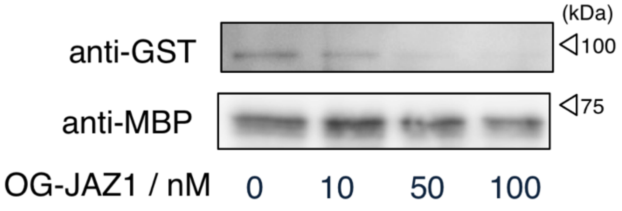

Supplementary Figure 3. Competitive inhibition of COI1-JAZ formation by OG-JAZ1. Pull-down of purified GST-COI1 (5 nM) with recombinant *E. coli*-expressed MBP-JAZ1 (full length, approximately 40 nM) and COR (**3**, 100 nM) in the absence or presence of OG-conjugated JAZ1 peptide (0–100 nM). GST-COI1 bound to MBP-JAZ proteins was pulled down with amylose resin and analyzed by immunoblotting. Goat HRP-conjugated anti-GST antibody was used for detection of GST-COI1. Rat anti-MBP antibody and goat HRP-conjugated rat IgG antibody were used to show the amounts of MBP-JAZ proteins as the input materials.

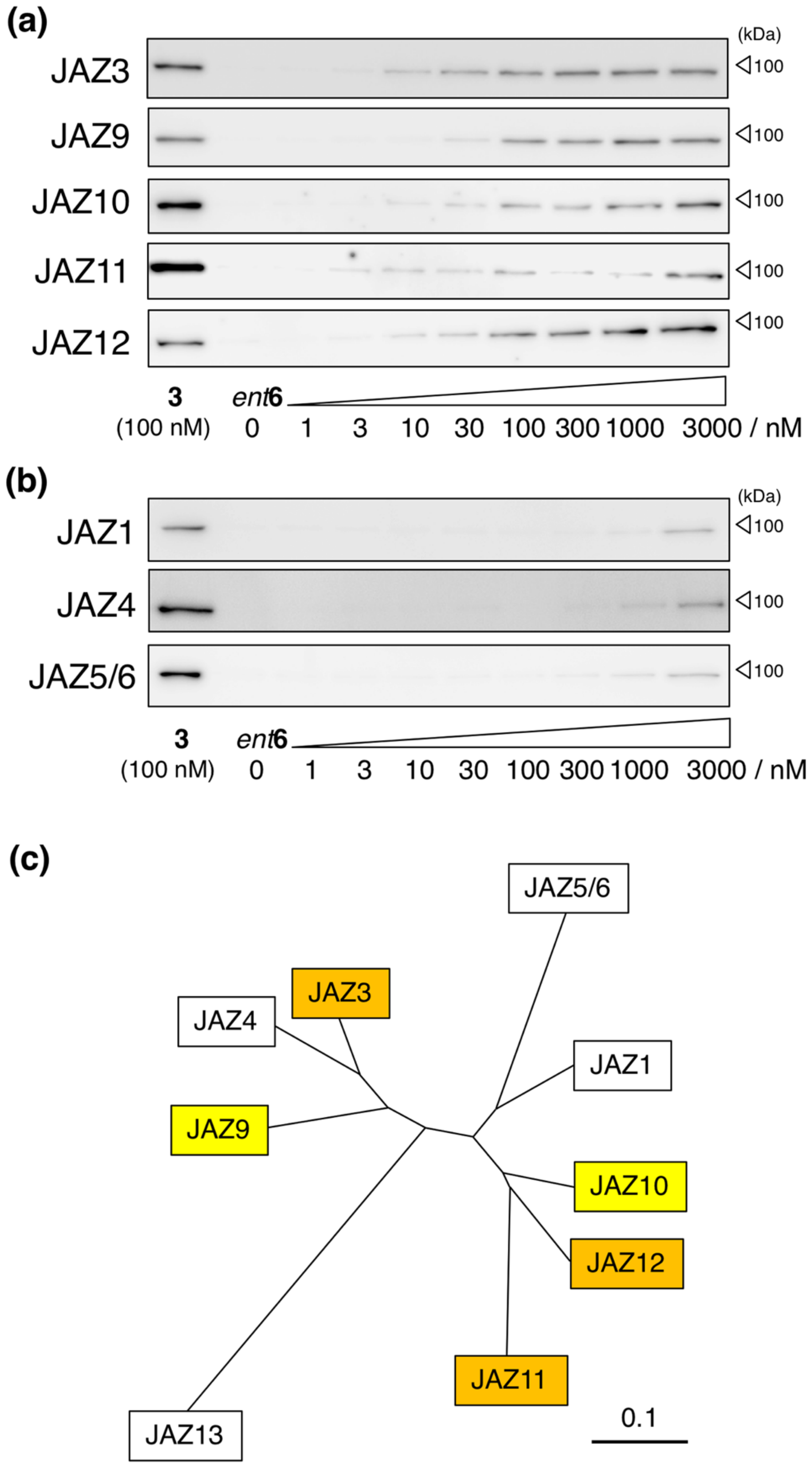

Supplementary Figure 4. Dose dependency of pull-down experiments with *ent***6**.

(**a**, **b**) Pull down assay of purified GST-COI1 (5 nM) with OG-conjugated JAZ peptides (**a**: JAZ3, 9, 10, 11 and 12, **b**: JAZ1, 4, 5/6; 10 nM) in the presence of **3** (100 nM) or *ent***6** (0–3000 nM). Goat HRP-conjugated anti-GST antibody was used for detection of GST-COI1. (**c**) Phylogenetic correlation of Jas motifs in JAZ subtypes evaluated by pull-down assays in Figure 2c. The subtypes of high affinity with *ent***6** (JAZ3, 11, 12) are shown in orange-colored boxes, and those of weak affinity (JAZ9 and 10) are shown in yellow-colored boxes.

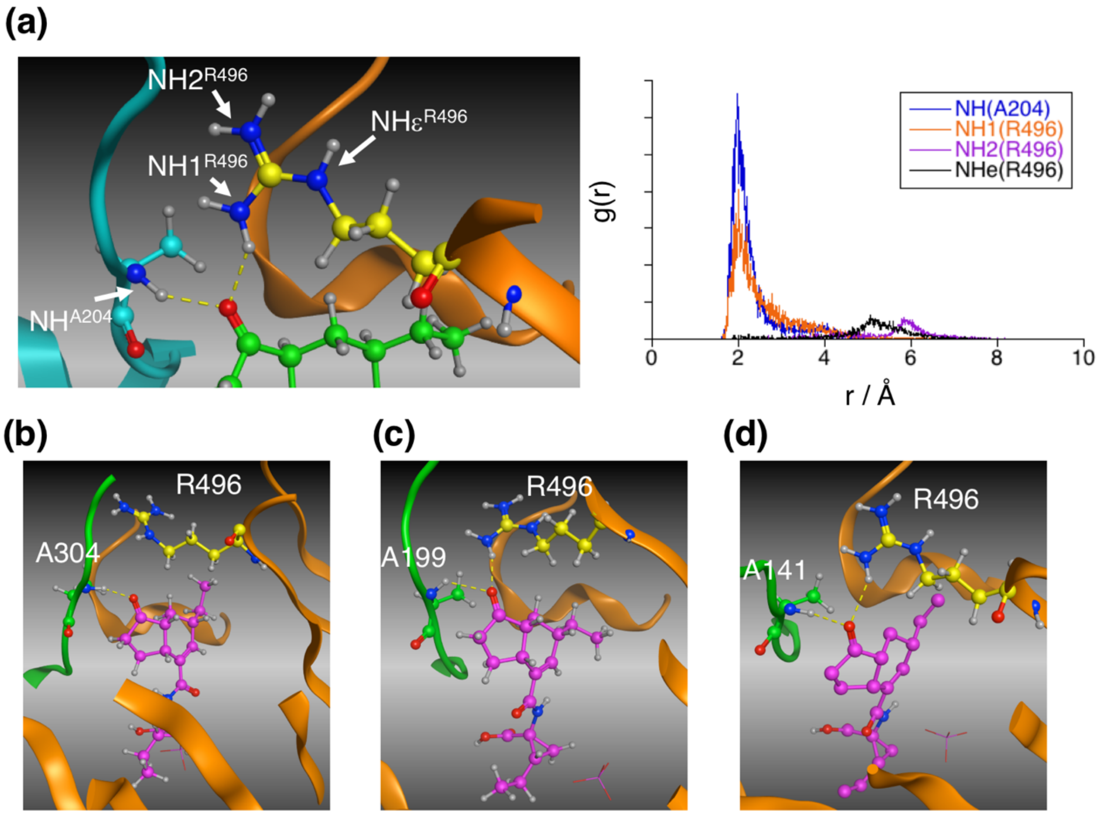

(e)

	Distance / Å	
	O-NH (Ala^{JAZ})	O-NH ($Arg496^{COI1}$)
COI1/**3**/JAZ1 (Fig. 3a)	1.96	2.01 (NH1)
COI1/*ent***6**/JAZ9 (Fig. 3b)	3.98	1.85 (NH1)
COI1/*ent***6**/JAZ10 (Fig. 3c)	2.03	1.94 (NH1)
COI1/*ent***6**/JAZ3 (Fig. S5b)	1.95	3.15 (NHε)
COI1/*ent***6**/JAZ11 (Fig. S5c)	2.15	1.89 (NH1)
COI1/*ent***6**/JAZ12 (Fig. S5d)	2.10	2.25 (NHε)

Supplementary Figure 5. *In silico* docking simulation of COI1- *ent***6**-JAZs.

(**a**) Left: Magnified image of the average structure for COI1/**3**/JAZ1, obtained by MD simulation. Three NH protons of $R496^{COI1}$ ($NH1^{R496}$, $NH2^{R496}$ and $NHε^{R496}$) and amide proton of $A204^{JAZ1}$ (NH^{A204}) are also shown; Right: The plots of the radial distribution function (g(r)) showing the distances between the ketone-oxygen atom in **3** and four nitrogen atoms of COI1 and JAZ1($NH1^{R496}$, $NH2^{R496}$, $NHε^{R496}$, and NH^{A204}) for 500 ns of MD time (right). MD simulation indicates that both of NH-proton in $NH1^{R496}$ and NH-

proton in A204^{JAZ1} mainly form hydrogen bonds. (**b**) The obtained average structure of COI1/*ent***6**/JAZ3 complex was constructed by *in silico* docking analysis and MD simulation. The hydrogen bond between the ketone of *ent***6** and A304^{JAZ3} (corresponding to A204^{JAZ1}) was indicated by a yellow dotted line. (**c**) The obtained average structure of COI1/*ent***6**/JAZ11 was constructed by *in silico* docking analysis and MD simulation. The hydrogen bond between the ketone of *ent***6** and R496^{COI1} was indicated by a yellow dotted line. (**d**) The obtained average structure of COI1/*ent***6**/JAZ12 complexed was constructed by *in silico* docking analysis and MD simulation. The hydrogen bond between the ketone of *ent***6** and A141^{JAZ3} (corresponding to A204^{JAZ1}) or R496^{COI1} was indicated by a yellow dotted line. (**e**) Summary of the average distances between the ketone-oxygen of **3** and NH-proton of R496^{COI1} or NH-proton of Ala^{JAZ} (COI1/**3**/JAZ1 and COI1/*ent***6**/JAZ9, JAZ10, JAZ3, JAZ11 or JAZ12), with 500 ns of MD time. Distances with possible hydrogen bond are indicated in blue color.

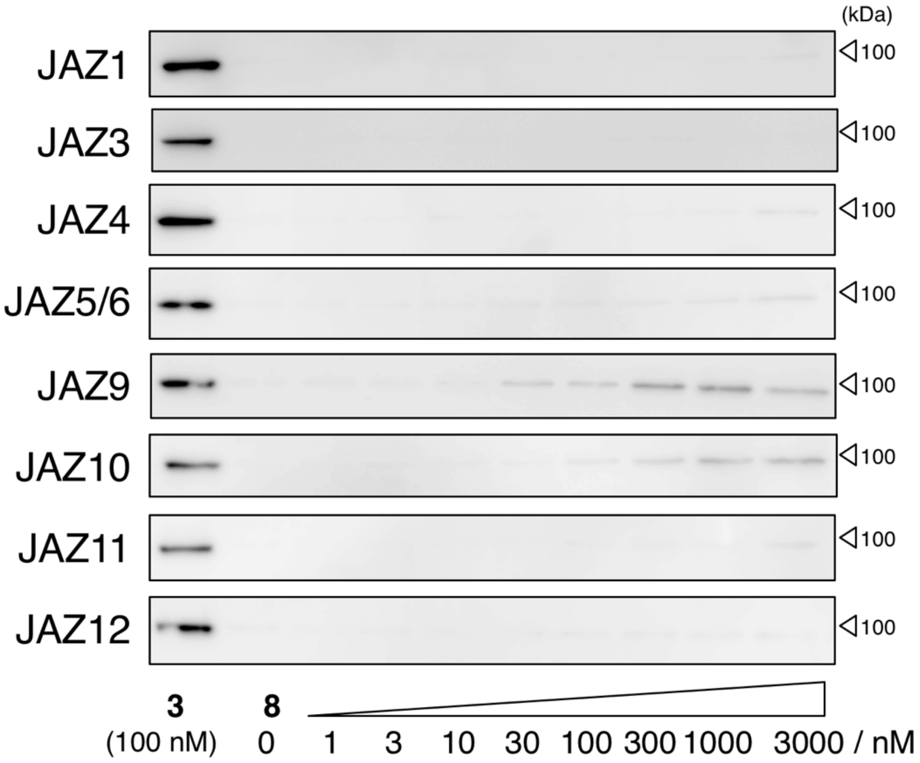

Supplementary Figure 6. Dose dependency of pull-down experiments with **8**.

Pull down assay of purified GST-COI1 (5 nM) with OG-conjugated JAZ peptides (JAZ1, 3, 4, 5/6, 9, 10, 11 and 12) in the presence of **8** (0–3000 nM) or **3** (100 nM). Goat HRP-conjugated anti-GST antibody was used for detection of GST-COI1.

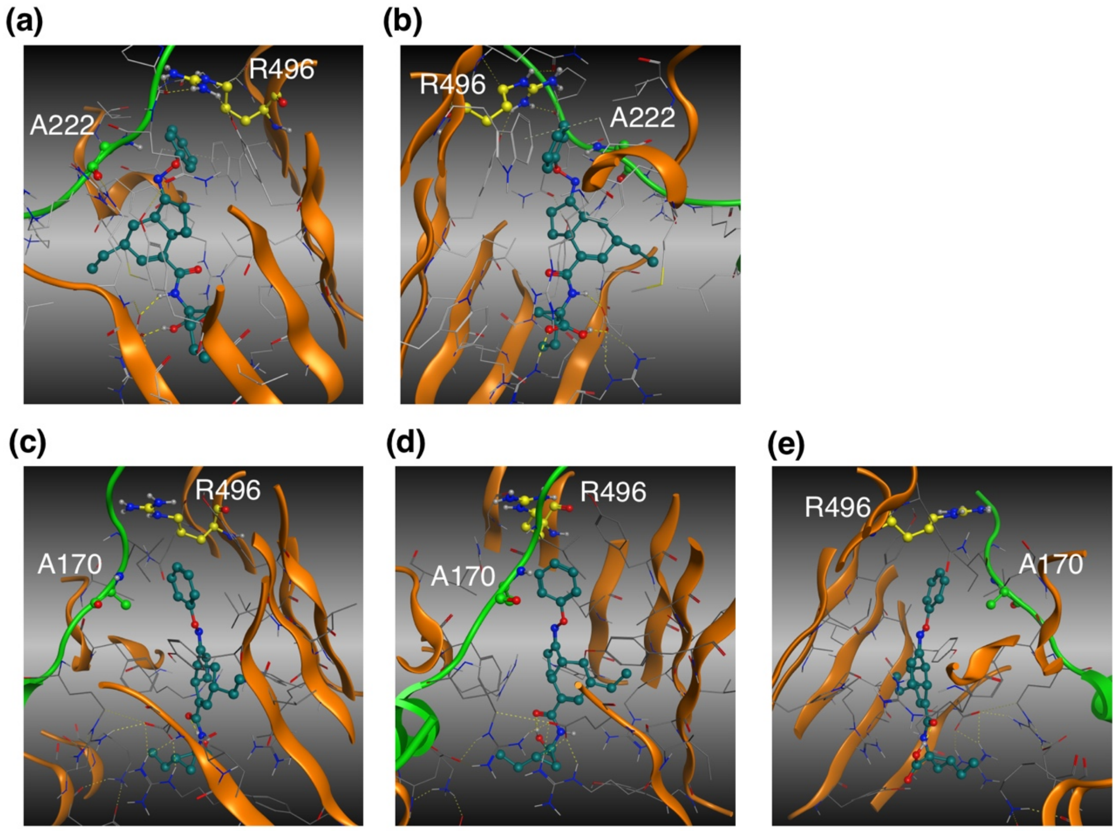

Supplementary Figure 7. *In silico* docking simulation of COI1-**8**-JAZ9/10.

The obtained average structures of COI1-**8**-JAZ9 (**a**, **b**) and COI1-**8**-JAZ10 (**c**-**e**) were constructed by *in silico* docking analyses and MD simulation.

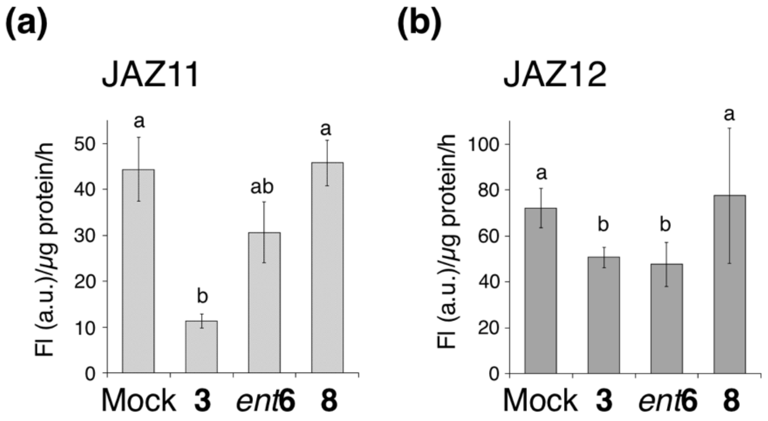

Supplementary Figure 8. GUS activities of compounds on JAZ11/JAZ12-GUS. Quantitative analyses of GUS activity in 20 roots of 4-d-old 35S:JAZ11-GUS (**a**) and 35S:JAZ12-GUS (**b**) plants (n = 4). Significant differences were evaluated by one-way ANOVA/Tukey HSD post hoc test (n = 4, p <0.01). Seedlings were pretreated for 30 min with or without ligand (**3**, *ent***6**, or **8**, 1 μM). Three independent replicates were used for the analyses and values represent mean ± s.d (supplementary methods).

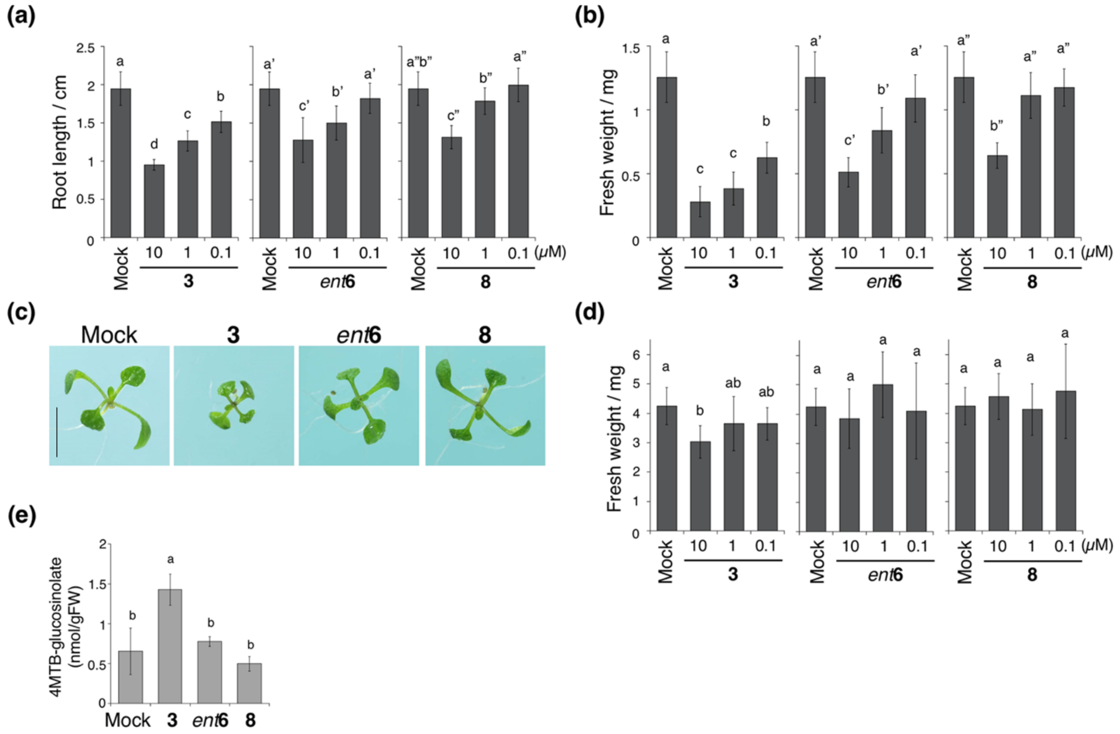

Supplementary Figure 9. Effects of compounds for *Arabidopsis* seedlings. (**a**, **b**) Dose-dependency of ligands (0.1–10 μM) in root length analyses (**a**) (n = 18) or fresh weight analyses for aerial part (**b**) (n = 15). Significant differences were evaluated by one-way ANOVA/Tukey HSD post hoc test (p <0.01). (**c, d**) The effects of the repetitive treatment of the compounds (**3**, *ent***6**, or **8** at 0.1–10 μM, first at 6th day and second at 9th day) in the aerial part of WT *Arabidopsis* seedlings grown for 9 d on 1/2 MS medium (see supplementary methods). Scale bar, 5 mm. The representative images of the aerial part treated with 1 μM of each compound (**c**) and fresh weights of aerial parts of the ligand-treated (0.1–10 μM) seedlings (**d**) (n = 5). Significant differences were evaluated by one-way ANOVA/HSD post hoc test (p <0.01). **(e)** 4-methylthiobutyl (4MTB)-glucosinolate accumulation in 6-d-old *Arabidopsis* seedlings (Col-0) with or without ligand (**3**, *ent***6**, or **8**, 1 μM) treatment for 1 d (n = 3). Significant differences were evaluated by one-way ANOVA/HSD post hoc test (p <0.01)

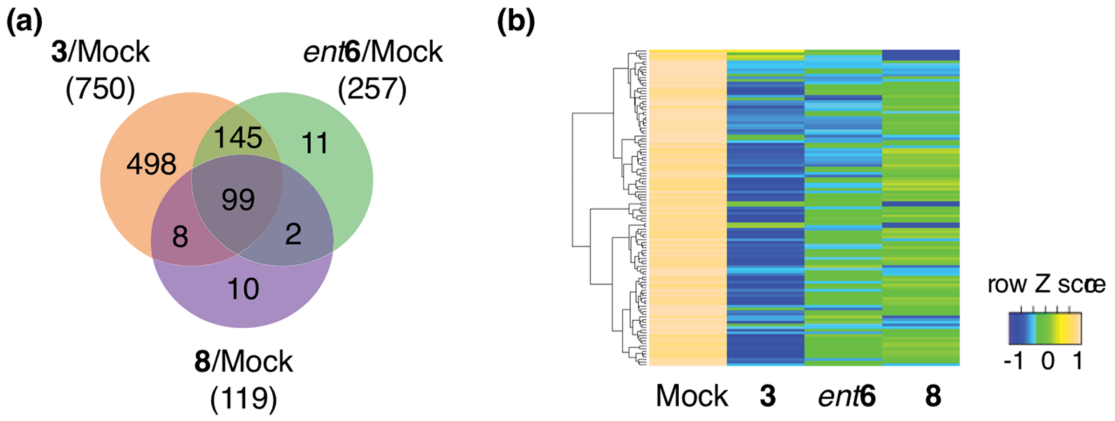

Supplementary Figure 10. Microarray analyses in *Arabidopsis thaliana* treated by f **3**, *ent***6**, or **8**. (**a**) Venn diagram indicating the number of genes at least 2.5 times down-regulated in response to different treatments. (**b**) Heat map illustrating changes in gene expression in response to different treatments. Genes with at least 2.5-fold decrease in expression by **8** are shown in blue ($p<0.05$, FDR followed by Tukey's HSD as post hoc test). The color represents the expression level of the gene. Yellow represents high expression, while blue represents low expression. The expression levels are continuously mapped on the color scale.

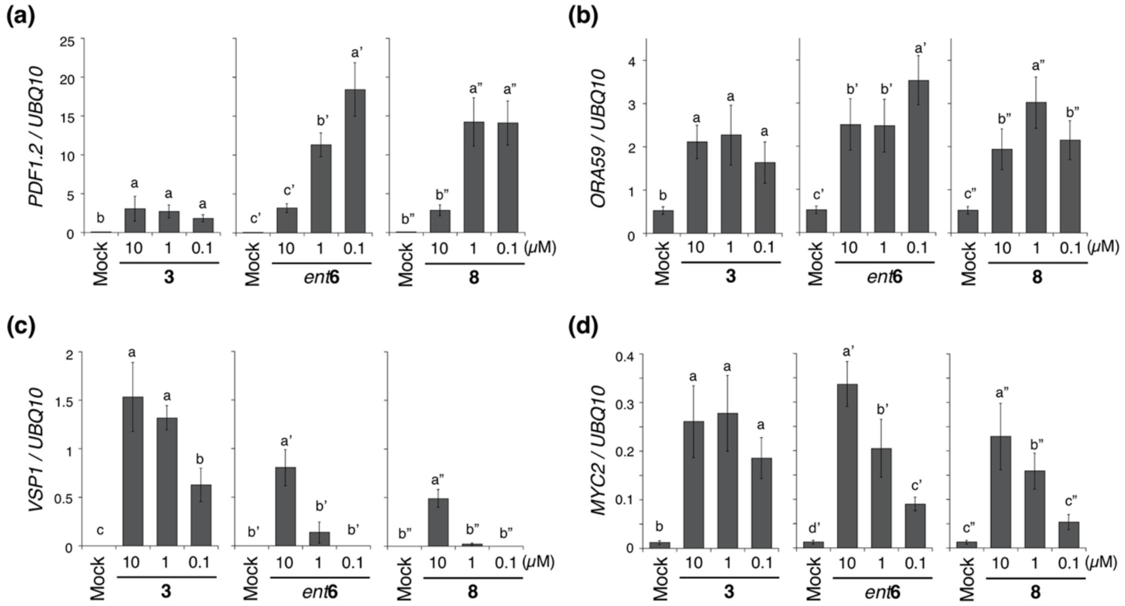

Supplementary Figure 11. Dose dependency of the gene expression analyses in WT *Arabidopsis* seedlings. Quantitative RT-PCR (qRT-PCR) analysis of JA-responsive gene expression in 6-day-old WT *Arabidopsis* seedlings treated with various concentrations of the ligands (**3**, *ent***6**, or **8**, 0.1 – 10 μM) treatment for 8 h (**a**, **c**) or for 2 h (**b**, **d**). The results are the mean with s.d. (n = 4). Significant differences were evaluated by one-way ANOVA/Tukey HSD post hoc test (p <0.05).

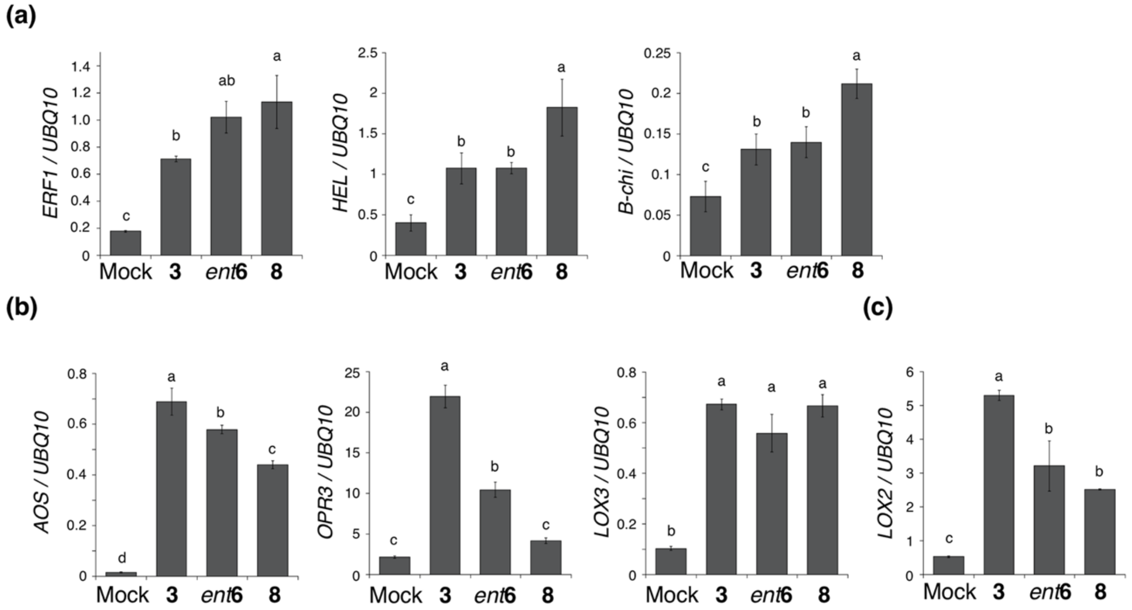

Supplementary Figure 12. Gene expression analyses in WT *Arabidopsis* seedlings treated by of **3**, *ent***6**, or **8**. (**a**) qRT-PCR analysis of the expression of *ERF1* and genes related to the JA-mediated plant defense responses (*HEL*, *B-chi*) in 6-d-old *Arabidopsis* WT seedlings (Col-0) with or without ligand (**3**, *ent***6**, or **8**, 1 μM) treatment for 8 h. (**b**) qRT-PCR analysis of the expression of JA-responsive genes including genes related to the biosynthesis of JA (*AOS*, *OPR3*, and *LOX3*) in 6-d-old *Arabidopsis* WT seedlings (Col-0) with or without ligand (**3**, *ent***6**, or **8**, 1 μM) treatment for 2 h. (**c**) qRT-PCR analysis of the expression of genes related to the biosynthesis of JA (*LOX2*) in 6-day-old *Arabidopsis* WT seedlings (Col-0) with or without ligand (**3**, *ent***6**, or **8**, 1 μM) treatment for 8 h. Results shown are the mean with s.d. (n = 3). Significant differences were evaluated by one-way ANOVA/Tukey HSD post hoc test ($p < 0.05$).

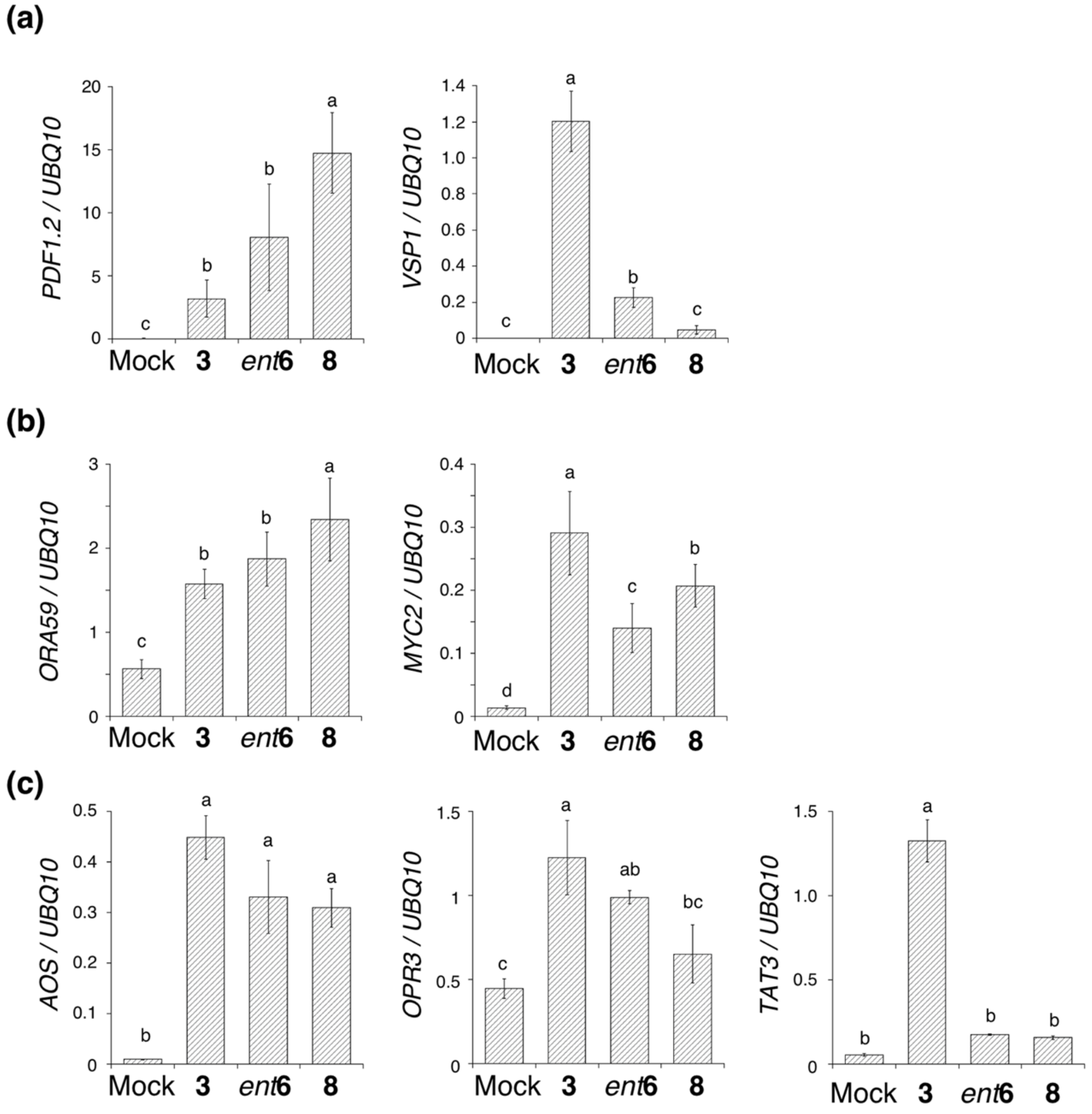

Supplementary Figure 13. JA-related gene expression analyses in *Arabidopsis* mutant seedlings (*jar1*) treated by **3**, *ent***6**, or **8**. (**a**) qRT-PCR analysis of the expression of JA-responsive genes (*PDF1.2*, and *VSP1*) in 6-d-old *Arabidopsis* mutant seedlings (*jar1*) with or without ligand (**3**, *ent***6**, or **8**, 1 μM) treatment for 8 h. (**b**) qRT-PCR analysis of the expression of JA-responsive genes of transcriptional factors (*ORA59*, and *MYC2*) in 6-d-old *Arabidopsis* mutant seedlings (*jar1*) with or without ligand (**3**, *ent***6**, or **8**, 1 μM) treatment for 2 h. **(c)** qRT-PCR analysis of the expression of JA-responsive genes (*AOS*, *OPR3*, and *TAT3*) in 6-d-old *Arabidopsis* mutant seedlings (*jar1*) with or without ligand (**3**, *ent***6**, or **8**, 1 μM) treatment for 2 h. Results shown are the mean with s.d. (n = 3). Significant differences were evaluated by one-way ANOVA/Tukey HSD post hoc test ($p < 0.05$).

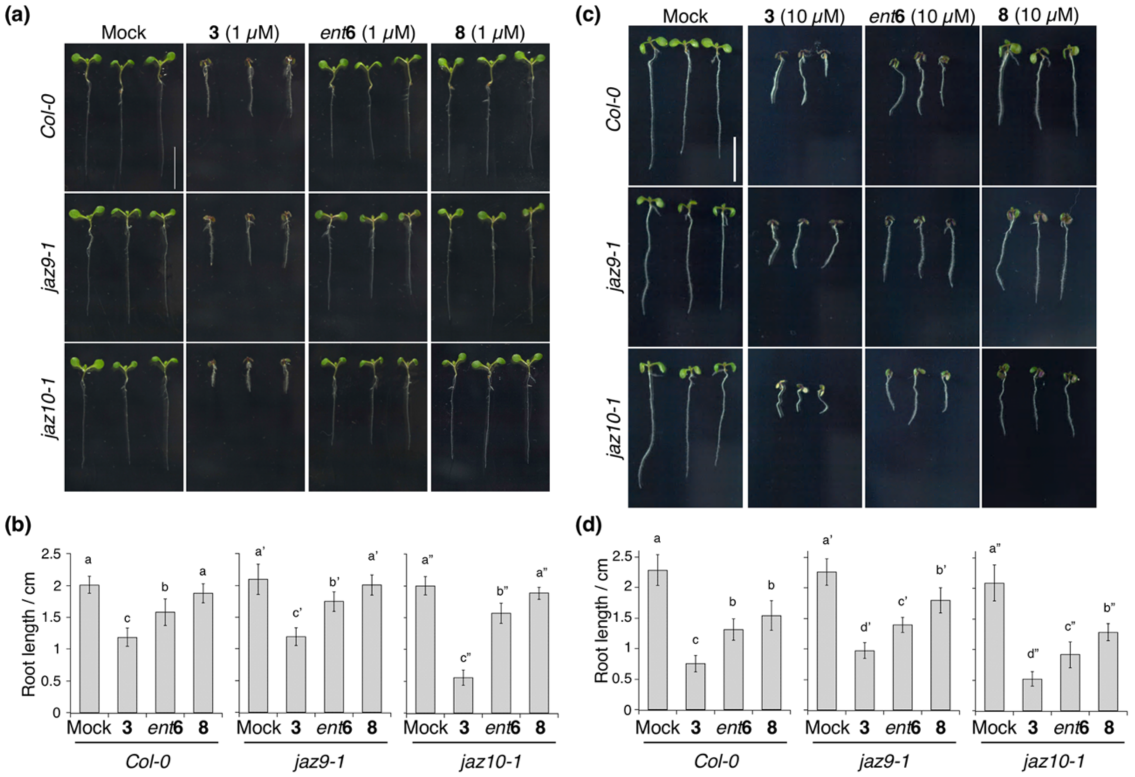

Supplementary Figure 14. Effects of **3**, *ent***6**, and **8** on the growth of *Arabidopsis* seedlings (*Col-0*, *jaz9-1*, *jaz10-1*). (**a**) *Arabidopsis* seedlings (*Col-0*, *jaz9-1*, *jaz10-1*) grown for 6 d on 1/2 MS medium supplemented with **3**, *ent***6**, or **8** (1 μM). Scale bar, 10 mm. (**b**) Quantitative analyses of root length or fresh weight of aerial part in the ligand-treated seedlings shown in (**a**). Results shown are the mean with s.d. (n = 17). (**c**) *Arabidopsis* seedlings (*Col-0*, *jaz9-1*, *jaz10-1*) grown for 6 d on 1/2 MS medium supplemented with **3**, *ent***6**, or **8** (10 μM). Scale bar, 10 mm. (**d**) Quantitative analyses of root length or fresh weight of aerial part in the ligand-treated seedlings shown in (**c**). Results shown are the mean with s.d. (n = 15). Significant differences were evaluated by one-way ANOVA/Tukey HSD post hoc test (p <0.01).

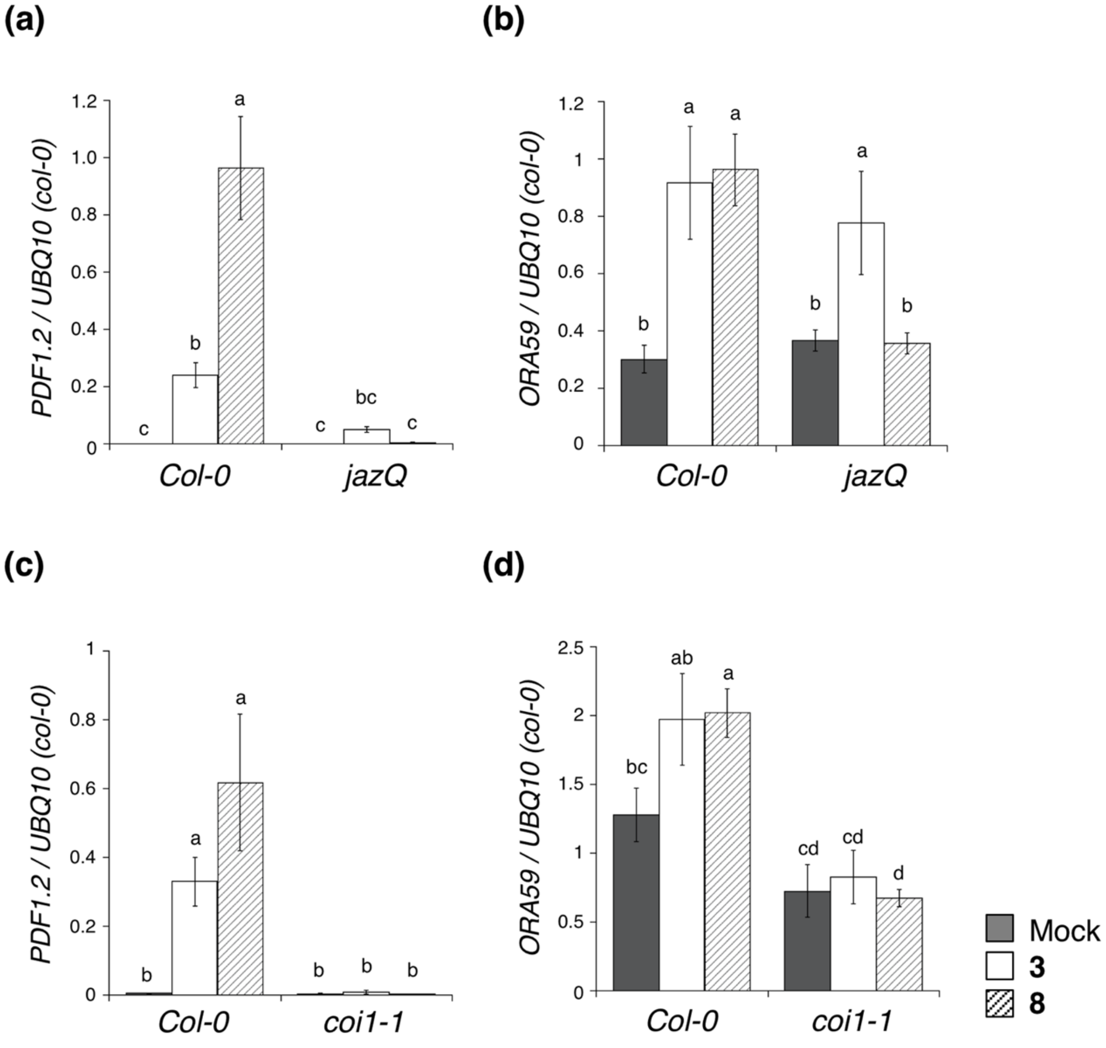

Supplementary Figure 15. JA-related gene expression analyses in *Arabidopsis* seedlings (*Col-0*, *jazQ* mutant, and *coi1-1* mutant) treated by of **3**, *ent***6**, or **8**. (**a, b**) qRT-PCR analysis of JA-responsive gene expression in 6-d-old WT (Col-0) or *jazQ Arabidopsis* seedlings with or without ligands (**3** or **8**, 1 μM) treatment (*PDF1.2* (**a**), *ORA59* (**b**)). Results shown are the mean with s.d. (n = ~~3~~ 4). (**c, d**) qRT-PCR analysis of JA-responsive gene expression in 6-d-old WT (Col-0) or *coi1-1 Arabidopsis* seedlings with or without ligands (**3** or **8**, 1 μM) treatment (*PDF1.2* (**c**), *ORA59* (**d**)). Results shown are the mean with s.d. (n = 4). Significant differences were evaluated by one-way ANOVA/Tukey HSD post hoc test ($p < 0.05$).

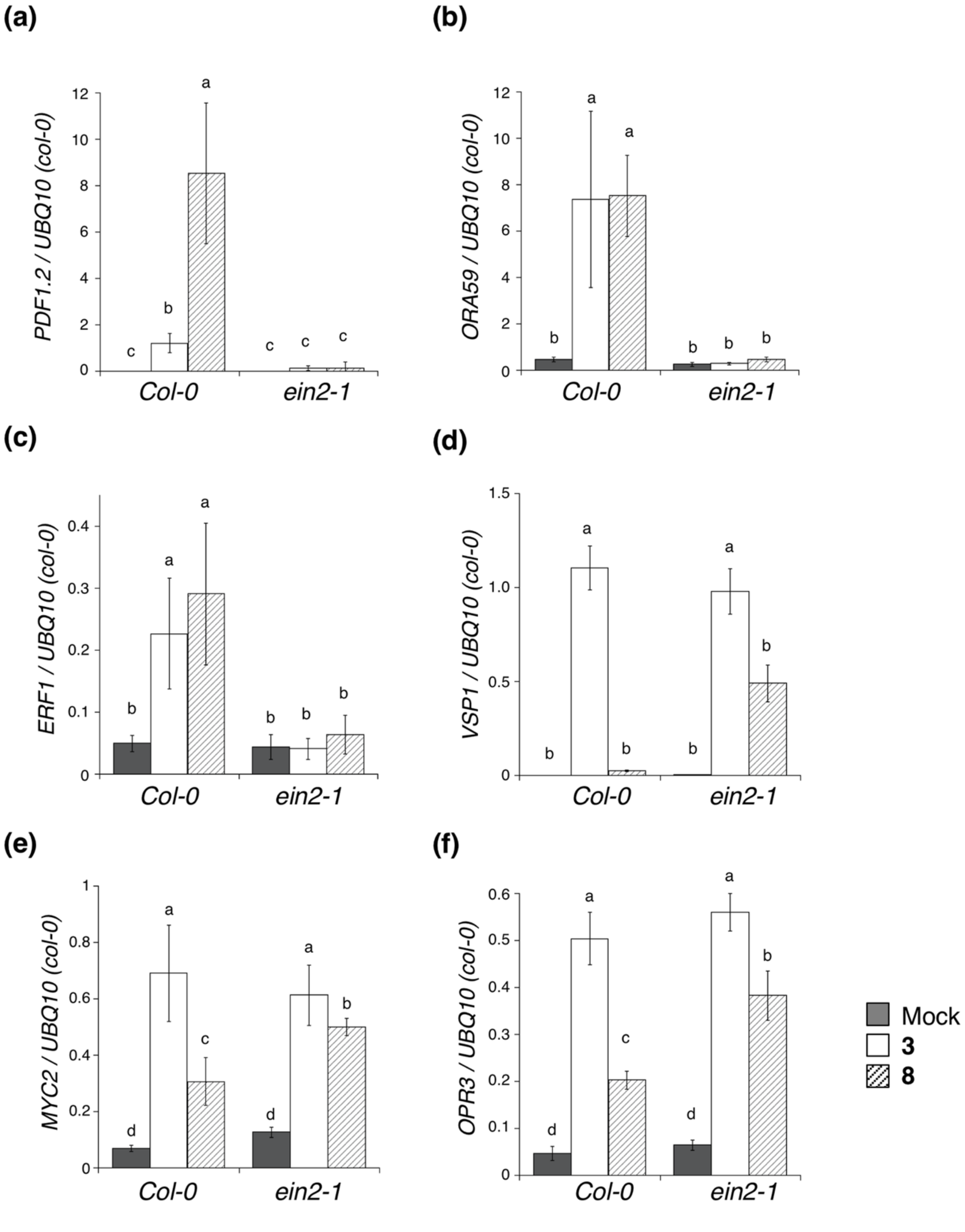

Supplementary Figure 16. JA-related gene expression analyses in *Arabidopsis* seedlings (*Col-0* and *ein2-1* mutant) treated by **3**, *ent***6**, or **8**. qRT-PCR analysis of JA-responsive gene expression in 6-d-old WT (Col-0) or *ein2-1 Arabidopsis* seedlings with or without ligands (**3** or **8**, 1 μM) treatment (*PDF1.2* (**a**), *ORA59* (**b**), *ERF1* (**c**), *VSP1* **(d)**, *MYC2* **(e)** or *OPR3* **(f)**). Results shown are the mean with s.d. (n = 4). Significant differences were evaluated by one-way ANOVA/Tukey HSD post hoc test ($p < 0.05$).

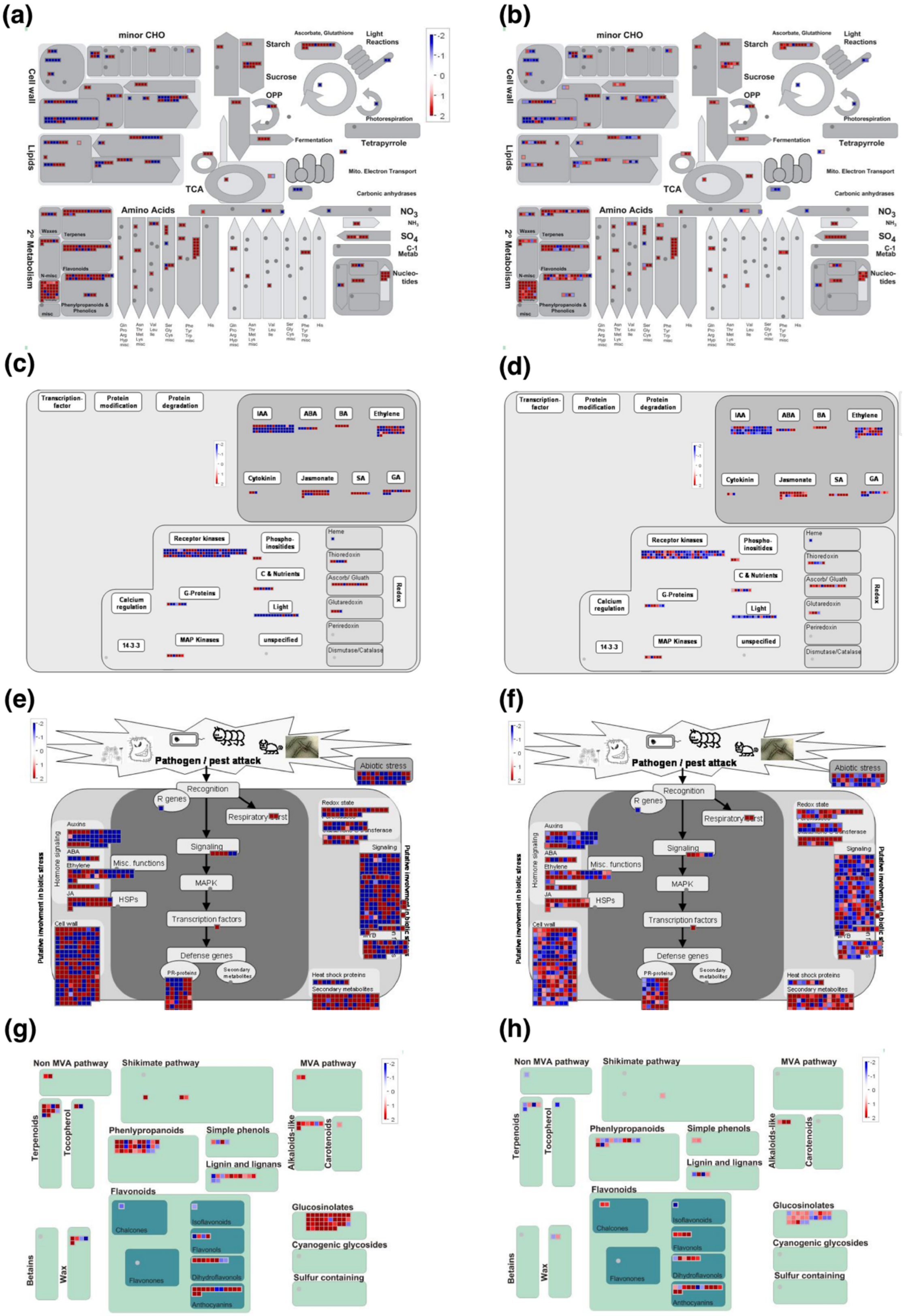
(a)
(b)
minor CHO
Cell wall
Starch
Sucrose
OPP
Ascorbate, Glutathione
Light Reactions
Photorespiration
Fermentation
Tetrapyrrole
Lipids
TCA
Mito. Electron Transport
Carbonic anhydrases
Amino Acids
2° Metabolism
Waxes
Terpenes
Flavonoids
N-misc
Phenylpropanoids & Phenolics
misc
Nucleo-tides
C-1 Metab
(c)
(d)
Transcription-factor
Protein modification
Protein degradation
IAA
ABA
BA
Ethylene
Cytokinin
Jasmonate
SA
GA
Receptor kinases
Phospho-inositides
C & Nutrients
G-Proteins
Light
Calcium regulation
14-3-3
MAP Kinases
unspecified
Heme
Thioredoxin
Ascorb/ Gluath
Glutaredoxin
Peroxiredoxin
Dismutase/Catalase
Redox
(e)
(f)
Pathogen / pest attack
Abiotic stress
Recognition
R genes
Respiratory burst
Signaling
MAPK
Transcription factors
Defense genes
PR-proteins
Secondary metabolites
Misc. functions
HSPs
Auxins
ABA
Ethylene
JA
Hormone signaling
Cell wall
Redox state
Heat shock proteins
Putative involvement in biotic stress
(g)
(h)
Non MVA pathway
Shikimate pathway
MVA pathway
Terpenoids
Tocopherol
Phenlypropanoids
Simple phenols
Lignin and lignans
Alkaloids-like
Carotenoids
Flavonoids
Chalcones
Isoflavonoids
Flavonols
Flavonones
Dihydroflavonols
Anthocyanins
Glucosinolates
Cyanogenic glycosides
Sulfur containing
Betains
Wax

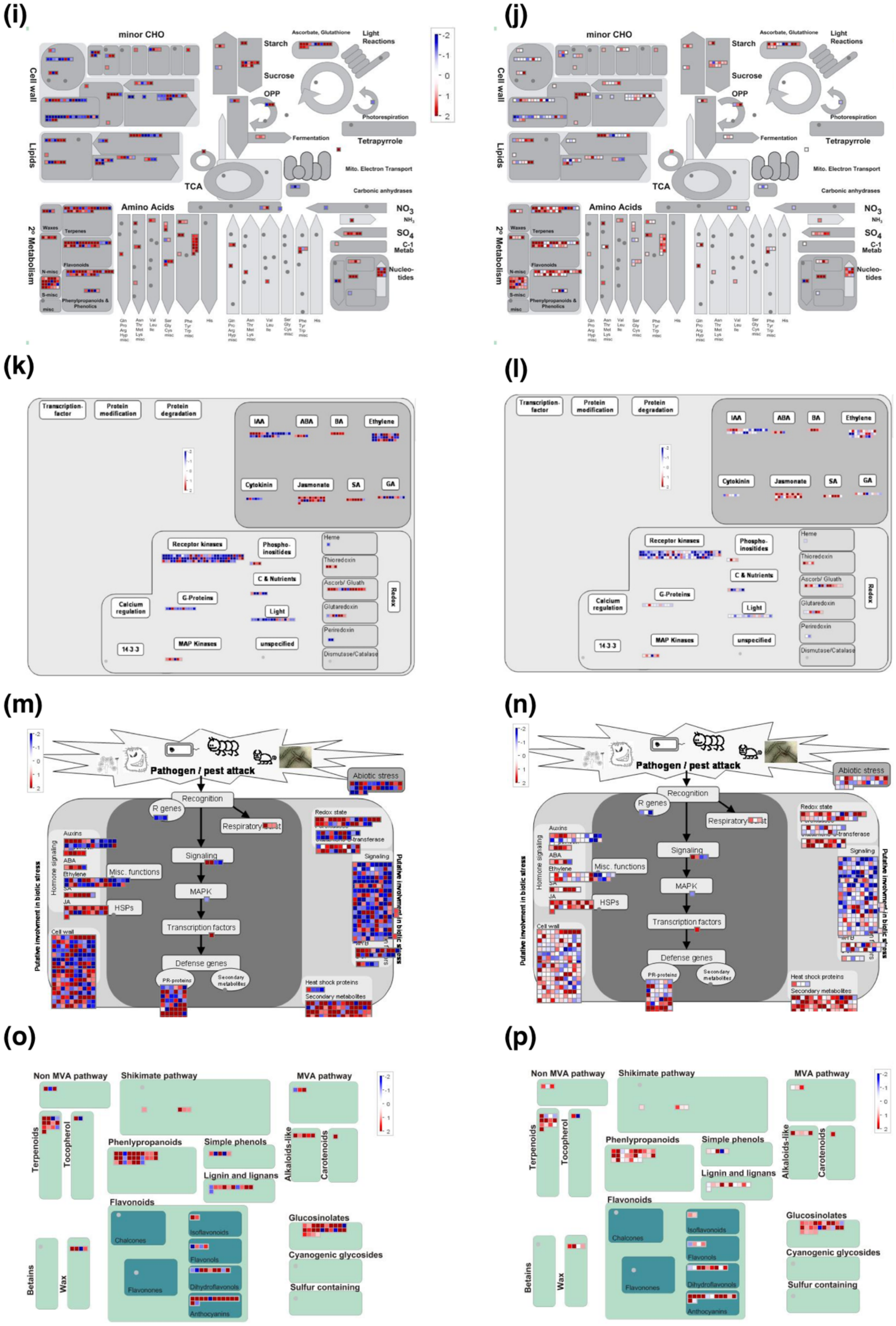
(i)
(j)
minor CHO
Cell wall
Lipids
Starch
Sucrose
OPP
Ascorbate, Glutathione
Light Reactions
Photorespiration
Fermentation
Tetrapyrrole
Mito. Electron Transport
Carbonic anhydrases
TCA
Amino Acids
NO_3
NH_3
SO_4
C-1 Metab
Nucleo-tides
2° Metabolism
Waxes
Terpenes
Flavonoids
N-misc
S-misc
misc
Phenylpropanoids & Phenolics
(k)
(l)
Transcription-factor
Protein modification
Protein degradation
IAA
ABA
BA
Ethylene
Cytokinin
Jasmonate
SA
GA
Receptor kinases
Phospho-inositides
C & Nutrients
G-Proteins
Calcium regulation
Light
14 3 3
MAP Kinases
unspecified
Heme
Thioredoxin
Ascorb/ Gluath
Glutaredoxin
Periredoxin
Dismutase/Catalase
Redox
(m)
(n)
Pathogen / pest attack
Abiotic stress
Recognition
R genes
Respiratory burst
Signaling
MAPK
Transcription factors
Defense genes
PR-proteins
Secondary metabolites
Hormone signaling
Auxins
ABA
Ethylene
SA
JA
Misc. functions
HSPs
Cell wall
Redox state
Signaling
Heat shock proteins
Secondary metabolites
Putative involvement in biotic stress
(o)
(p)
Non MVA pathway
Shikimate pathway
MVA pathway
Terpenoids
Tocopherol
Phenlypropanoids
Simple phenols
Lignin and lignans
Alkaloids-like
Carotenoids
Flavonoids
Chalcones
Isoflavonoids
Flavonols
Flavonones
Dihydroflavonols
Anthocyanins
Glucosinolates
Cyanogenic glycosides
Sulfur containing
Betains
Wax

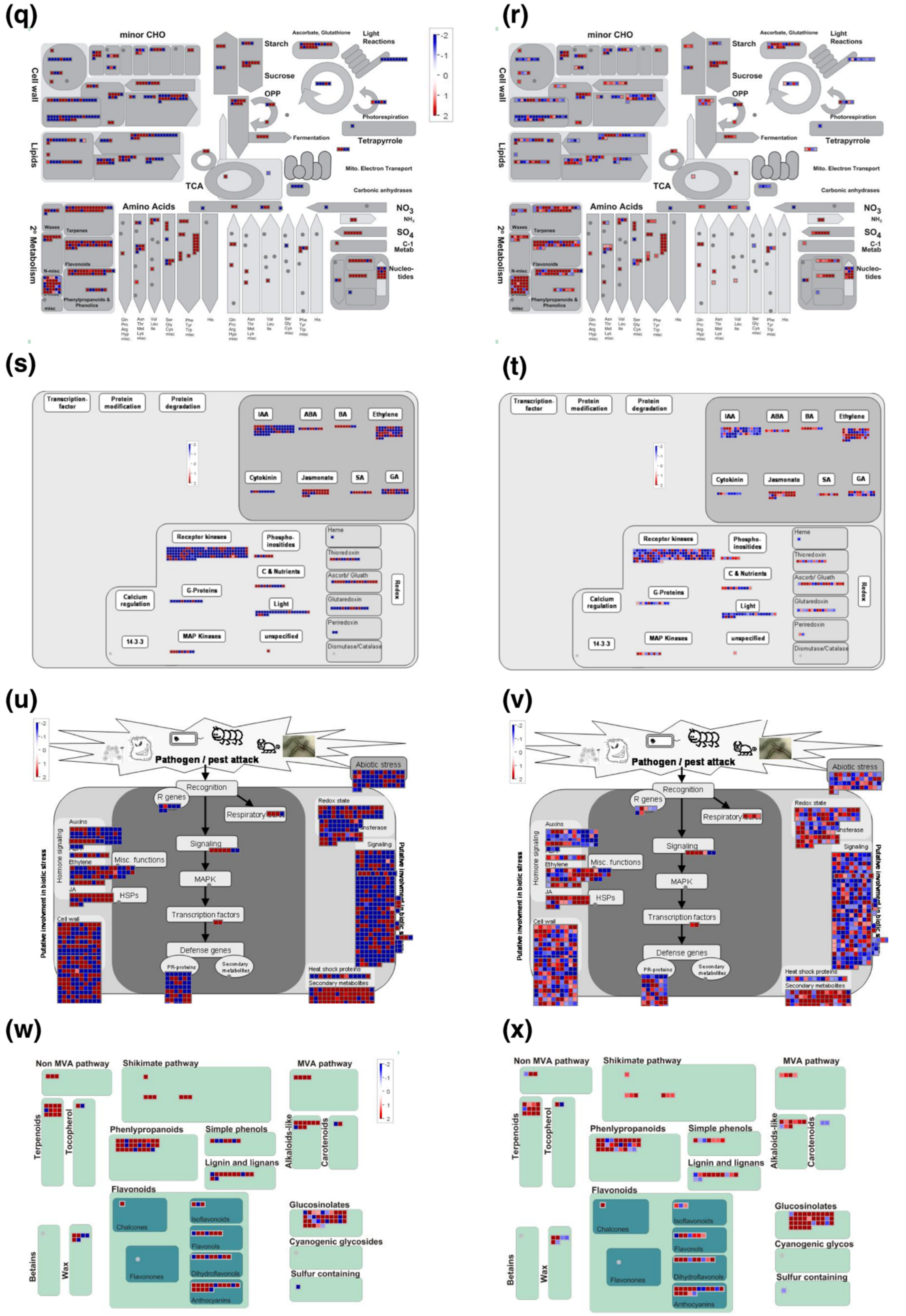

Supplementary Figure 17. Overview of microarray analyses in *Arabidopsis* seedlings treated by **3** or **8**. **(a, b)** Metabolism overview, **(c, d)** regulation overview, **(e, f)** biotic

stress overview and **(g, h)** secondary metabolism in WT. Changes in metabolic related genes in response to treatment with **3 (a, c, e, g)** and **8 (b, d, f, h)**. **(i, j)** Metabolism overview, **(k, l)** regulation overview, **(m, n)** biotic stress overview and **(o, p)** secondary metabolism in *jaz9* mutant. Changes in metabolic related genes in response to treatment with **3 (i, k, m, o)** and **8 (j, l, n, p)**. **(q, r)** Metabolism overview, **(s, t)** regulation overview, **(u, v)** biotic stress overview and **(w, x)** secondary metabolism in *jaz9* mutant. Changes in metabolic related genes in response to treatment with **3 (q, s, u, w)** and **8 (r, t, v, x)**.

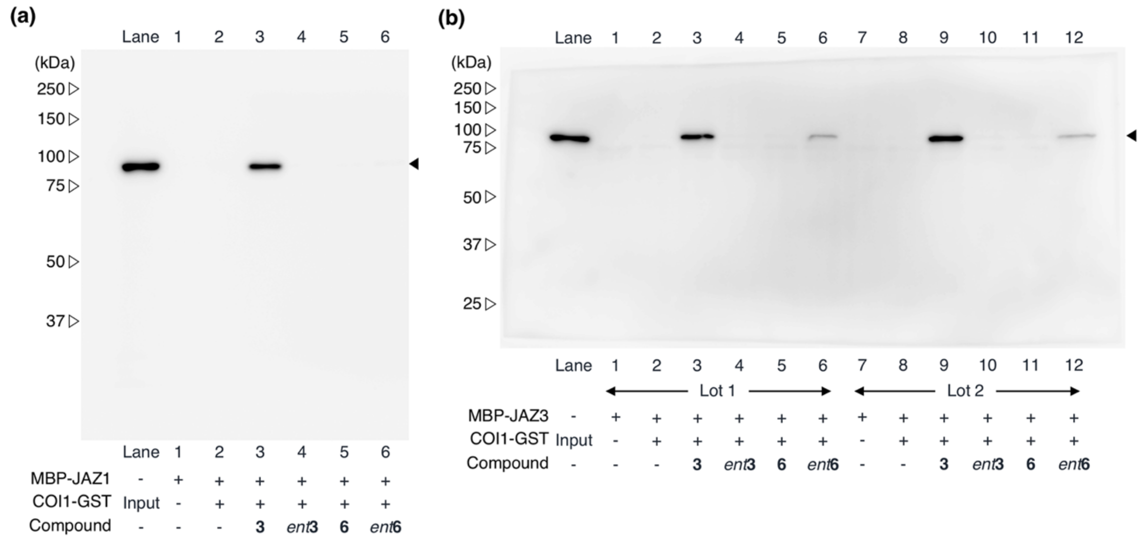

Supplementary Figure 18. Uncropped blots of **Figure 1de**. Pull down assay of purified GST-COI1 with recombinant proteins expressed in *E. coli*, including (**a**) MBP-JAZ1 (full length) and (**b**) MBP-JAZ3 (full length, two independent results were shown in one blot), in the presence of COR derivatives. GST-COI1 bound to MBP-JAZ proteins was pulled down with amylose resin and analyzed by immunoblotting. Goat HRP-conjugated anti-GST antibody was used to detect GST-COI1 (black triangles).

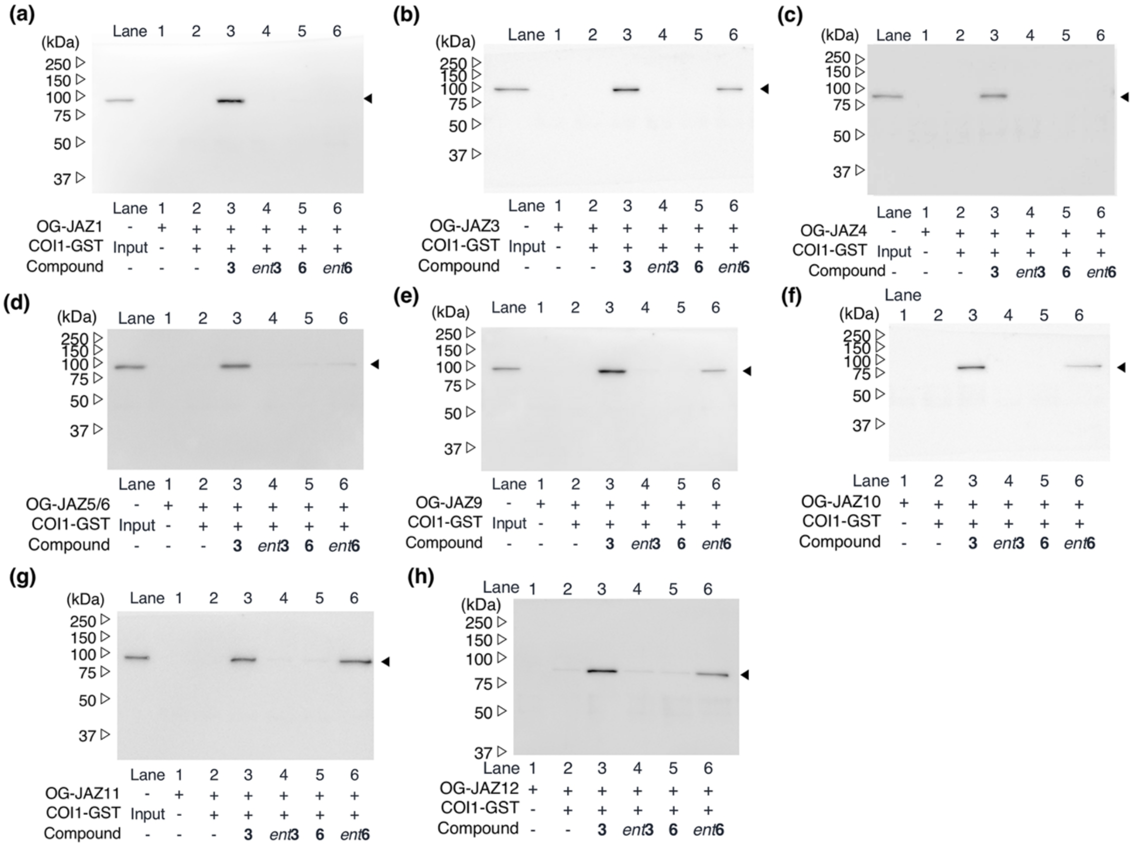

Supplementary Figure 19. Uncropped blots of **Figure 2c**. Pull-down assay of purified GST-COI1 with each OG-conjugated JAZ peptide (**a**: JAZ1, **b**: JAZ3, **c**: JAZ4, **d**: JAZ5/6, **e**: JAZ9, **f**: JAZ10, **g**: JAZ11, **h**: JAZ12) in the presence of stereoisomers of (+)-COR (**3**, *ent***3**, **6**, or *ent***6**). Goat HRP-conjugated anti-GST antibody was used to detect GST-COI1 (black triangles).

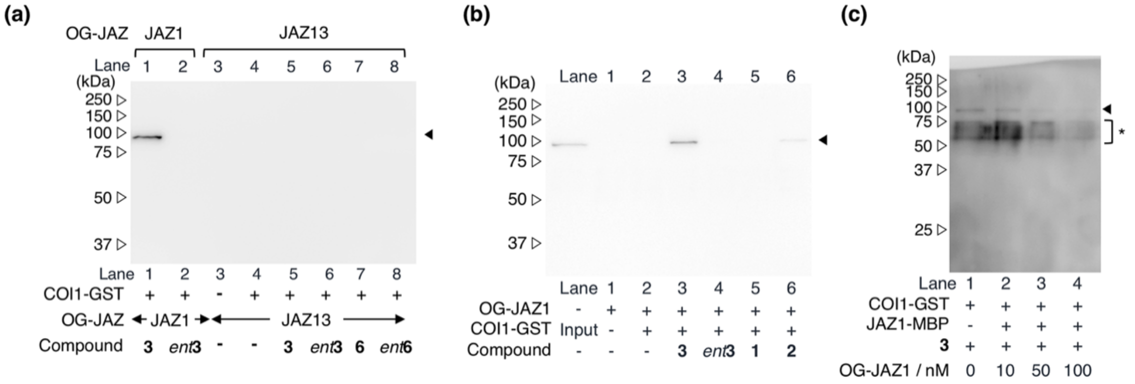

Supplementary Figure 20. Uncropped blots of **Figure 2de** (**a**, **b**) and **Supplementary Figure 3** (**c**). **(a)** Pull-down assay of purified GST-COI1 with OG-conjugated JAZ13 peptide in the presence of **3**, *ent***3**, **6** or *ent***6**, as shown in **Figure 2d**. **(b)** Pull-down assay of purified GST-COI1 with OG-conjugated JAZ1 peptide in the presence of **3**, *ent***3**, **1** or **2**, as shown in **Figure 2e**. **(c)** Dose dependency of OG-JAZ1 for pull-down experiments of COI1 with full-length JAZ1, as shown in **Supplementary Figure 3**. Pull-down of purified GST-COI1 with recombinant *E. coli*-expressed MBP-JAZ1 (full length) and COR (**3**) in the absence or presence of OG-conjugated JAZ1 peptide (*asterisk indicate the non-specific staining). Goat HRP-conjugated anti-GST antibody was used to detect GST-COI1 (black triangles).

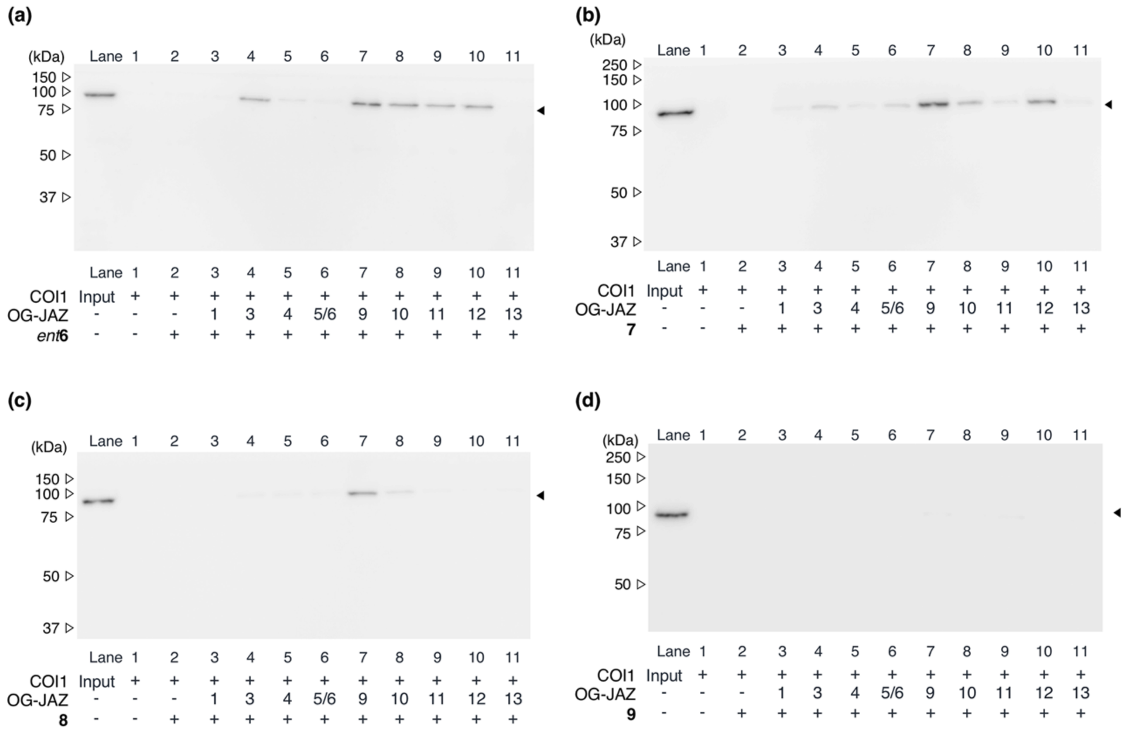

Supplementary Figure 21. Uncropped blots of **Figure 3e**. Pull-down assay of purified GST-COI1 with all OG-conjugated JAZ peptides in the presence of *ent***6** (**a**), **7** (**b**), **8** (**c**), or **9** (**d**) (500 nM). HRP-conjugated anti-GST antibody was used to detect GST-COI1. Goat HRP-conjugated anti-GST antibody was used to detect GST-COI1 (black triangles).

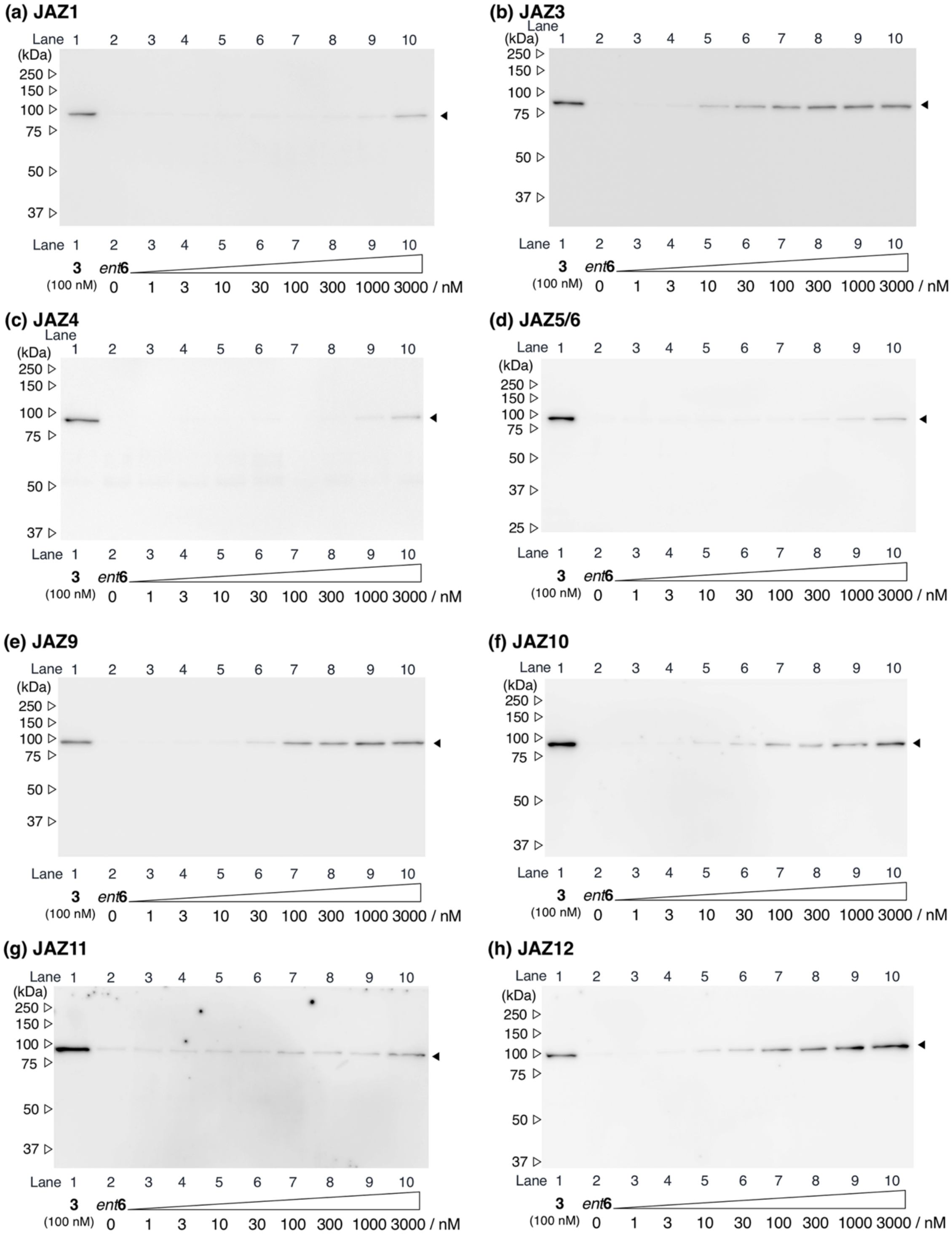

Supplementary Figure 22. Uncropped blots of **Supplementary Figure 4a**. Dose dependency of pull-down experiments of COI1 with OG-conjugated JAZs and *ent*6. Pull down assay of purified GST-COI1 with OG-conjugated JAZ peptides (**a**; JAZ1, **b**; JAZ3, **c**; JAZ4, **d**; JAZ5/6, **e**; JAZ9, **f**; JAZ10, **g**; JAZ11, **h**; JAZ12) in the presence of **3** (100 nM) or *ent*6 (0–3000 nM).

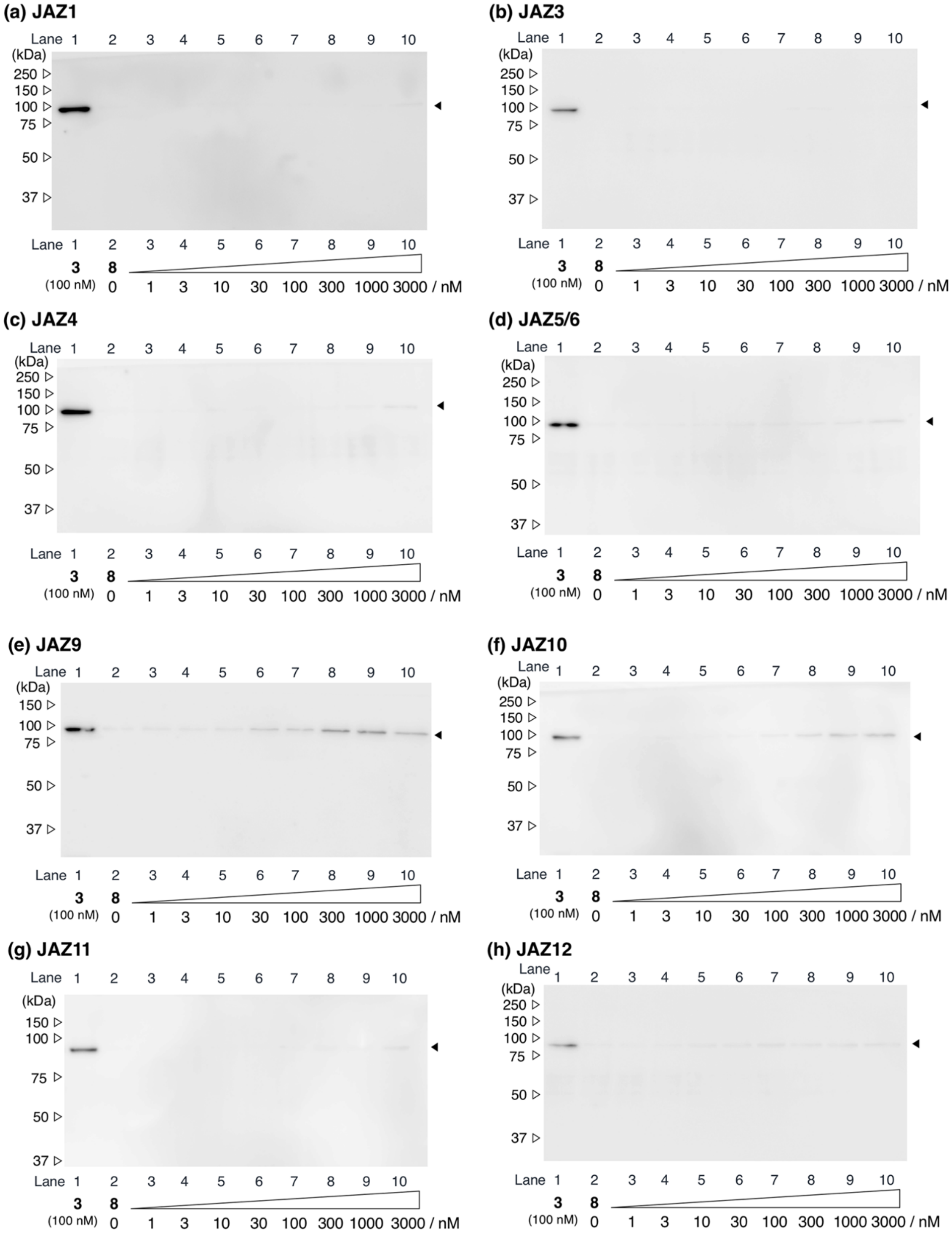

Supplementary Figure 23. Uncropped blots of **Supplementary Figure 6**. Dose dependency of pull-down experiments of COI1 with OG-conjugated JAZs and **8**. Pull down assay of purified GST-COI1 with OG-conjugated JAZ peptides (**a**; JAZ1, **b**; JAZ3, **c**; JAZ4, **d**; JAZ5/6, **e**; JAZ9, **f**; JAZ10, **g**; JAZ11, **h**; JAZ12) in the presence of **3** (100 nM) or **8** (0–3000 nM).

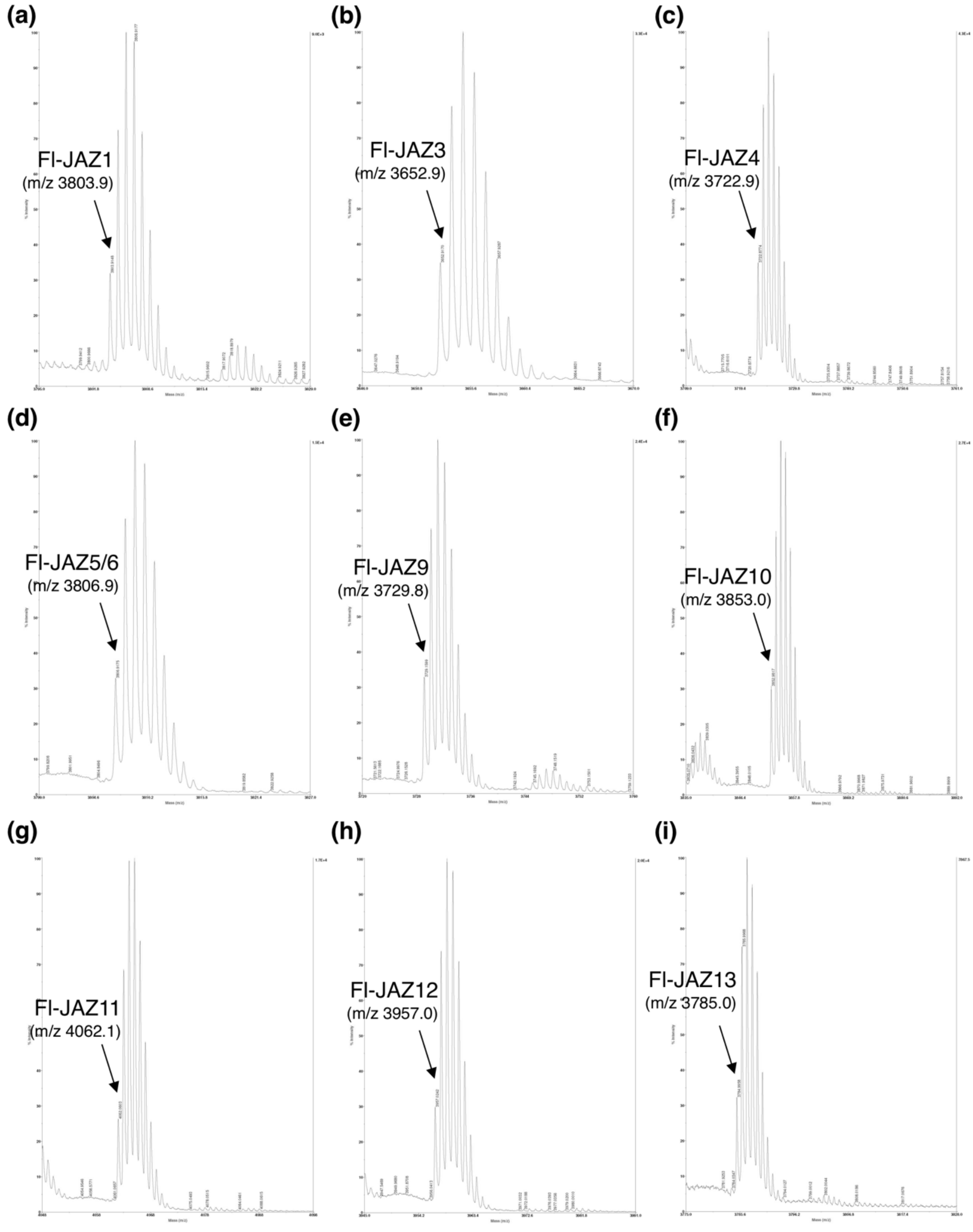

Supplementary Figure 24. MALDI-TOF MS spectra of OG-conjugated peptides; **(a)** OG-JAZ1, **(b)** OG-JAZ3, **(c)** OG-JAZ4, **(d)** OG-JAZ5/6, **(e)** OG-JAZ9, **(f)** OG-JAZ10, **(g)** OG-JAZ11, **(h)** OG-JAZ12, and **(i)** OG-JAZ1~~2~~ 13.

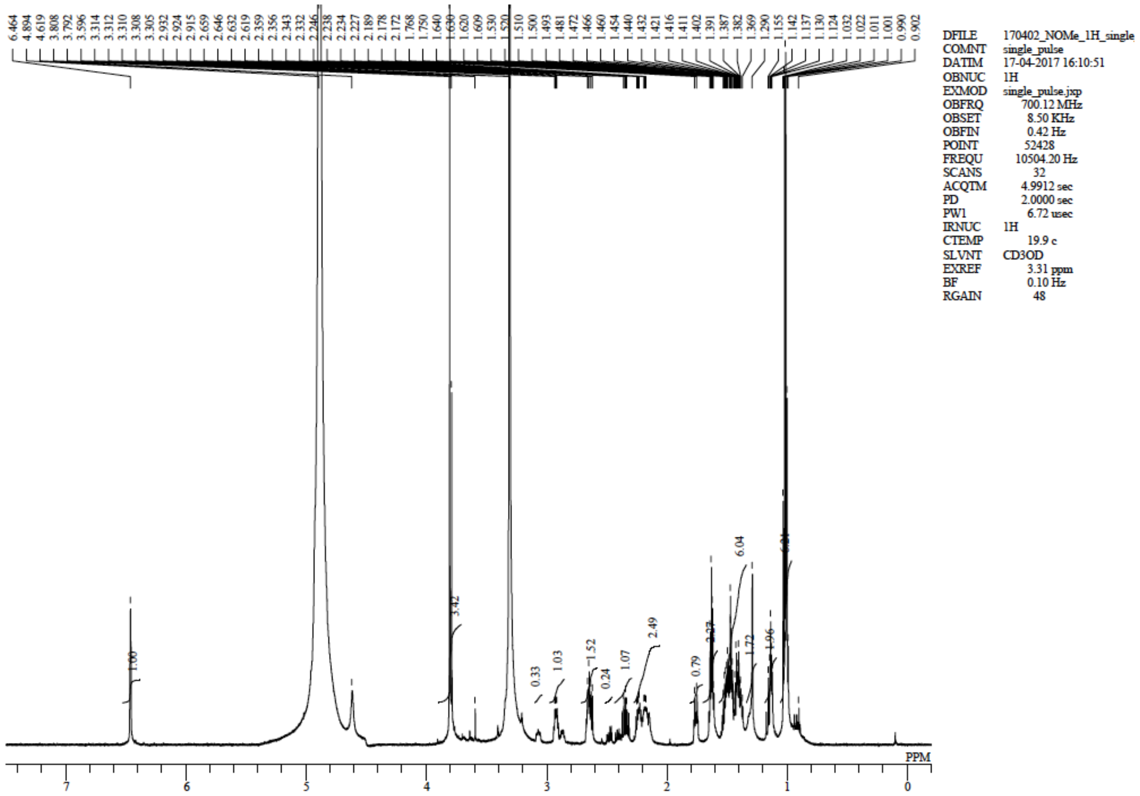

Supplementary Figure 25. ^{1}H NMR spectrum of compound **7**.

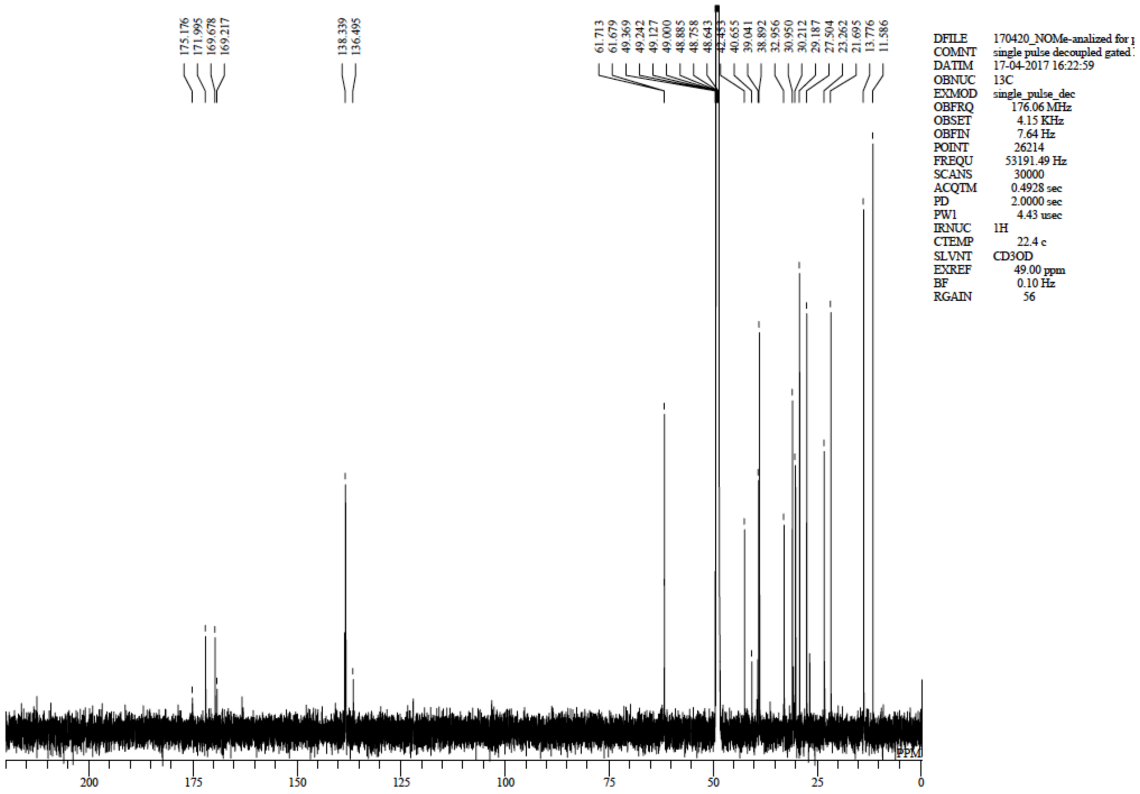

Supplementary Figure 26. ^{13}C NMR spectrum of compound **7**.

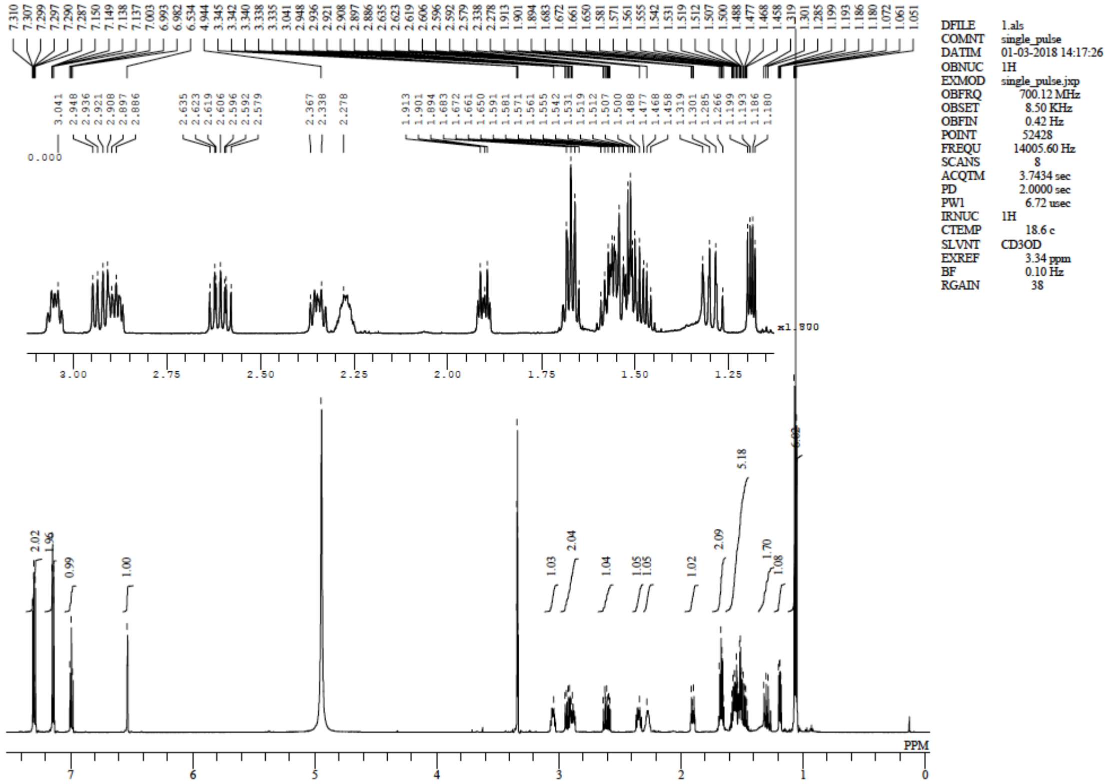

Supplementary Figure 27. ^{1}H NMR spectrum of compound **8**.

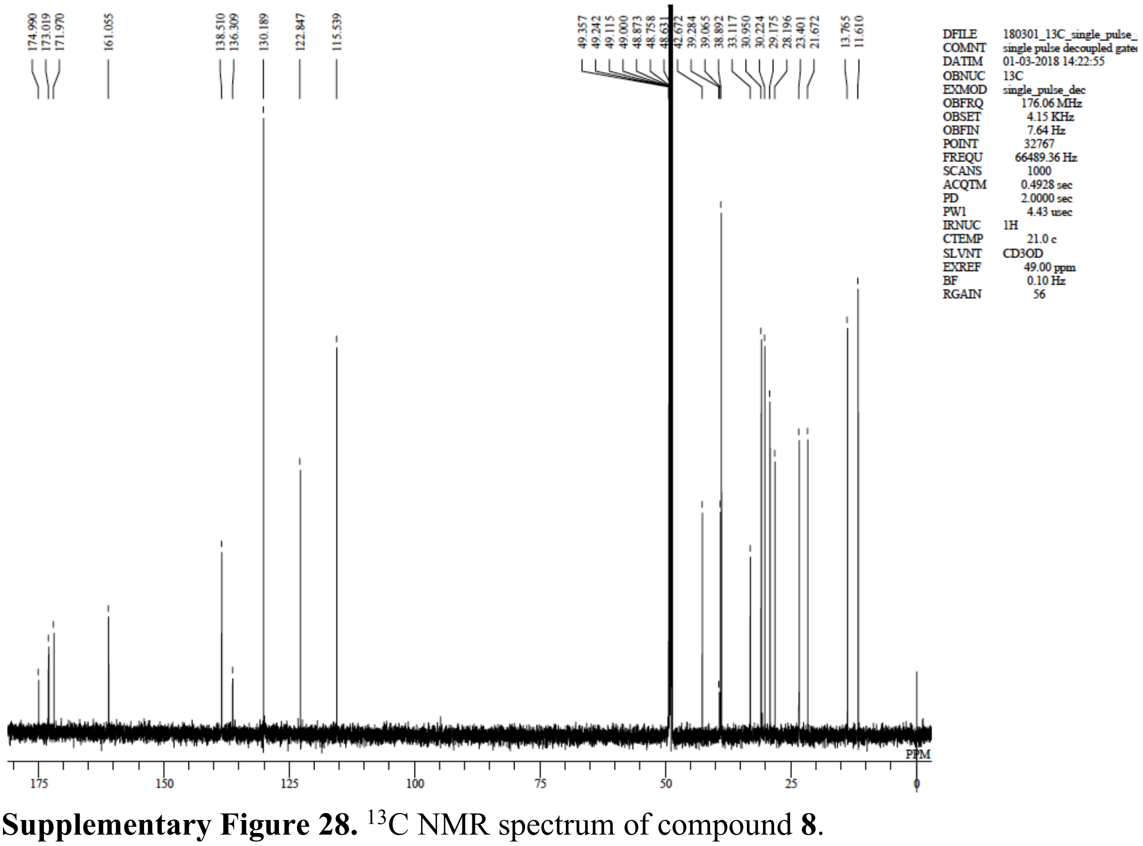

Supplementary Figure 28. ^{13}C NMR spectrum of compound **8**.

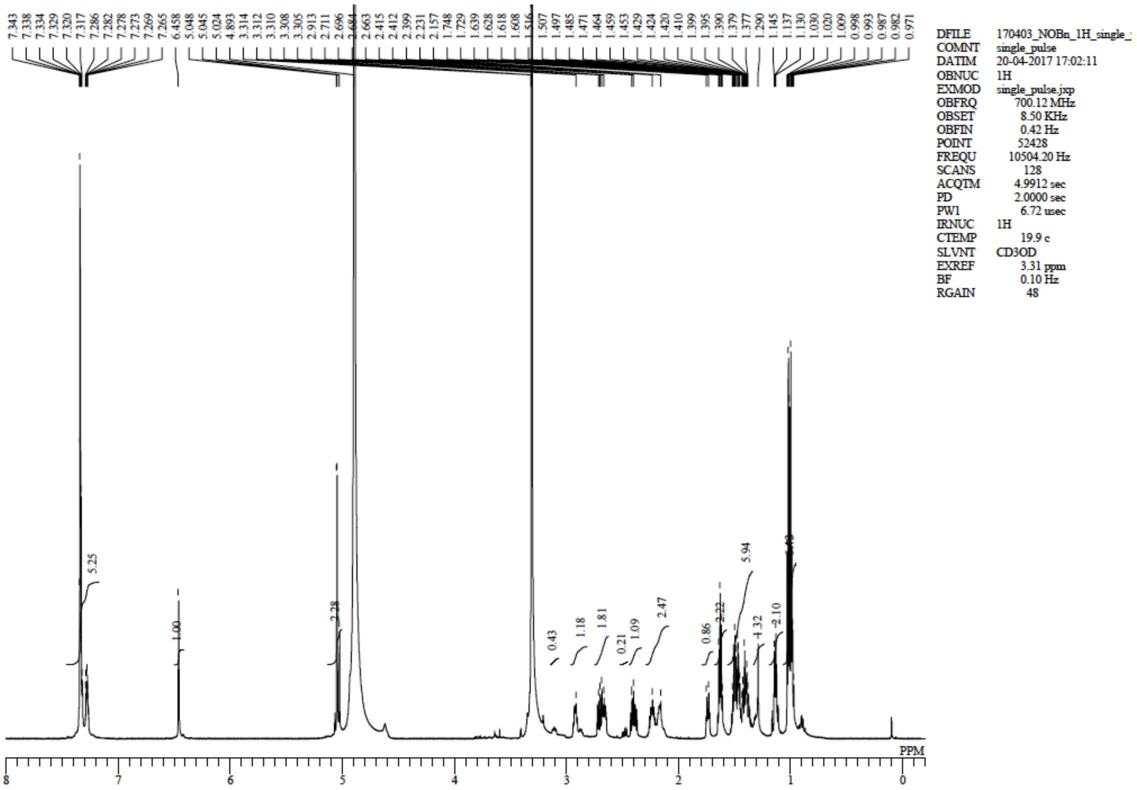

Supplementary Figure 29. ^{1}H NMR spectrum of compound **9**.

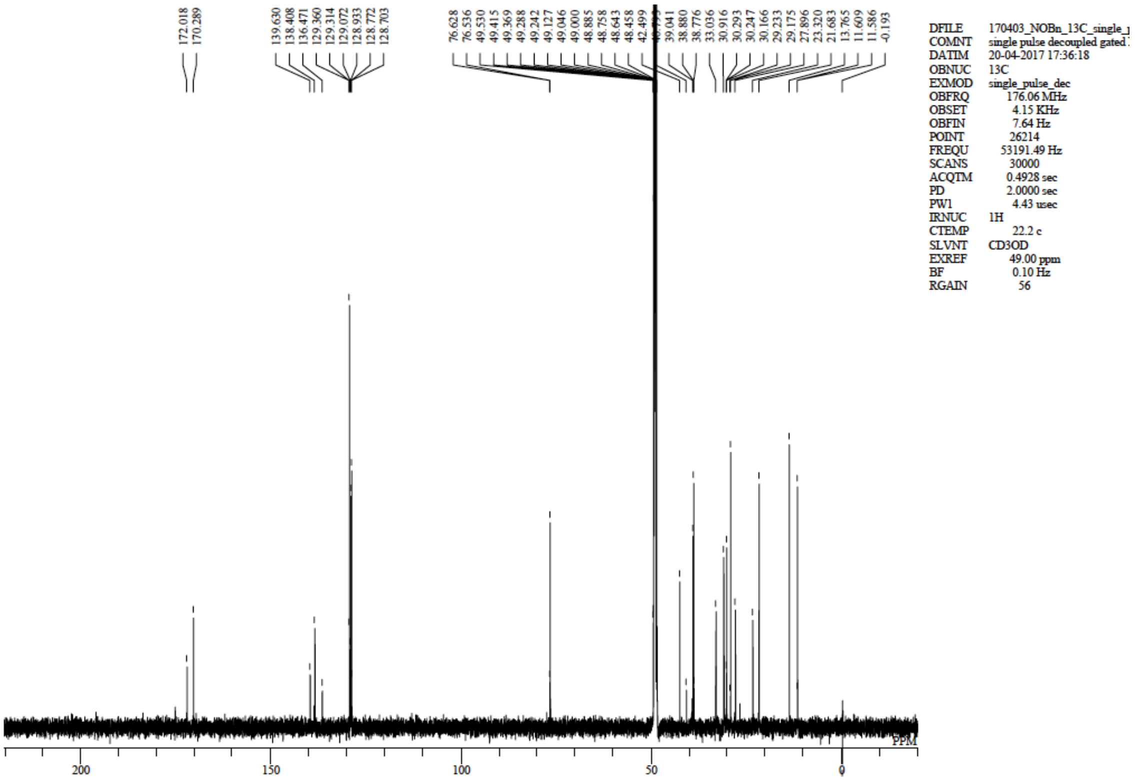

Supplementary Figure 30. ^{13}C NMR spectrum of compound **9**.

Supplementary Table 1. Summary of the gene sequences of all primers for qRT-PCR.

Protein TIFY 10A	5’ GTCTTCAAACCCTCAAAC 3’
(*JAZ1*: AT1G19180)	5’ AGCATGAAGATAGGAGCTT 3’
Allene oxide synthase	5’ CTCCGTTAATTTCTCGTC 3’
(*AOS*: AT5G42650)	5’ GCAGCAACAGATTATACAAC 3’
Oxophytodienoate reductase 3	5’ CGGTTCAAGATTGATGGAGA 3’
(*OPR3*: AT2G06050)	5’ CGATTATCAAACTCAGAGGC 3’
Lipoxygenase 3	5’ CACTGCAATTCACAAGCAACC 3’
(*LOX3*: AT1G17420)	5’ CAAAGGAGGAATCGGAGAAGC 3’
Lipoxygenase 2	5’ CAAGGATGCTGGCCTCTTAC 3’
(*LOX2*: AT3G45140)	5’ TCGTCTCGTAACCATGAAAATC 3’
Tyrosine aminotransferase 3	5’ AAGCTGAAGGCCGAGGATGTGTAT 3’
(*TAT3*: AT2G24850)	5’ TCCCGGCCTTGGAAGTAGAATGTT 3’
Plant defensin 1.2	5’ ATGTCTTCCCAGCACACAAG 3’
(*PDF1.2*: AT5G44420)	5’ GTTACTCATAGAGTGACAGAGAC 3’
Vegetative storage protein 1	5’ CAATGGGCTGATTTGGTTG 3’
(*VSP1*: AT5G24780)	5’ GGATACAAGGGGACAATGCC 3’
Ethylene-responsive	5’ TCATGTTCTTGATAATCTCTGCTTC 3’
transcription factor ERF094	5’ CACGGCCATCACATCTCTTC 3’
(*ORA59*: AT1G06160)	
Transcription factor MYC2	5’ ATCTATACGCAAGAACAGC 3’
(*MYC2*: AT1G32640)	5’ GACCCCATAACTTTCTAAAC 3’
Ethylene-response factor1	5’ TTAATTCAGTCCCCATTCTC 3’
(*ERF1*: AT3G23240)	5’ CCTCTGAGTCGTTCTCGTTG 3’
Pathogenesis-related 4	5’ CCCAATGAGCTCATTGCCACAGT 3’
(*HEL*: AT3G04720)	5’ CGGTTACTGTGGTCACACCGCGG 3’
Pathogenesis-related 3	5’ AGTCCTTCCCCGCTTTTG 3’
(*B-chi*: AT3G12500)	5’ GGTTCACAGTAGTCTGAAGC 3’
Polyubiquitin 10	5’ GGCCTTGTAATAATCCCTGATGAATAAG 3’
(*UBQ10*: AT4G05320)	5’ AAAGAGATAACAGGAACGGAAACATAGT 3’

Supplementary Methods

Synthesis of epitope-conjugated JAZ peptides.

Cysteine-introduced JAZ peptides were purchased from BioGate Co., Ltd (Gifu, Japan). The following protocol was shown for OG-JAZ1 peptide as a representative example: Purified JAZ1 peptide (0.5 mg) was dissolved in 500 μL of 1 : 1 solution of phosphate buffered saline (pH 7.4) and DMSO containing tris (2-carboxyethyl) phosphine (TCEP, 10 mM). The solution was mixed with Oregon Green (OG) 488 maleimide (0.3 mg, 5 eq) and incubated at 37 ºC for 12 h. The reaction mixture was purified by HPLC using a Develosil ODS-HG-5 column (Φ 4.6 × 250 mm) with linear gradient (CH_3CN (0.05% TFA):H_2O (0.05% TFA) = 20:80 (5 min) to 50:50 (35 min)) to afford fluorophore-conjugated JAZ peptide. In case of OG-JAZ13, the peptide was prepared by microwave-assisted solid phase synthesis with NovaSyn® TGA resin (90 μm) using Initiator$^+$ Alstra (Biotage Ltd, North Carolina, US), and the solid phase-peptide was vortexed with 5-carboxy-OG (3 eq), *N,N,N',N'*-tetramethyl-*O*-(1*H*-benzotriazol-1-yl)uranium hexafluorophosphate (HBTU, 5 eq), 1-hydroxybenzotriazol monohydrate (HOBt•H_2O) (5 eq), and DIPEA (5 eq) in dry DMF at r.t. for 2 h. The resulting peptide was deprotected with TFA solution at r.t. for 1.5 h. The reaction mixture was purified by HPLC. After lyophilization, each conjugated JAZ peptide was dissolved in sterilized water to prepare the stock solution. The concentration of the solution was calculated from UV-vis absorption spectrum using molar extinction coefficient of OG (ε491 = 76,000 $M^{-1}cm^{-1}$ in 0.1N NaOH aq.).[S5] The purity of these peptides was confirmed by HPLC analyses (Supplementary Figure 2), and were characterized by MALDI-TOF MS analyses (Supplementary Figure 24) as follows;

OG-JAZ1: m/z $[M+H]^+$ calcd for 3803.9, found 3803.9

OG-JAZ3: m/z $[M+H]^+$ calcd for 3652.9, found 3652.9

OG-JAZ4: m/z $[M+H]^+$ calcd for 3722.9, found 3722.9

OG-JAZ5/6: m/z $[M+H]^+$ calcd for 3806.9, found 3806.9

OG-JAZ9: m/z $[M+H]^+$ calcd for 3729.8, found 3728.8

OG-JAZ10: m/z $[M+H]^+$ calcd for 3852.9, found 3853.0

OG-JAZ11: m/z $[M+H]^+$ calcd for 4062.0, found 4062.1

OG-JAZ12: m/z $[M+H]^+$ calcd for 3957.0, found 3957.0

OG-JAZ13: m/z $[M+H]^+$ calcd for 3785.0, found 3785.0

Synthesis of compounds 7–9.

Compound **7**: *O*-methylhydroxylamine hydrochloride (14.1 mg, 159 μmol) was added to a stirred solution of *ent***6**[1] (1.7 mg, 5.3 μmol) in dry pyridine (0.5 mL). The mixture was stirred at r.t. for 12 h; then, *O*-methylhydroxylamine hydrochloride (1.5 mg, 18 μmol) was added, and the solution stirred at r.t. for an additional 2 d. The mixture was diluted with distilled water and extracted with EtOAc. The combined organic layers were washed with distilled water and brine; dried over anhydrous Na_2SO_4; and concentrated *in vacuo*. The residue was purified by HPLC using a Develosil ODS-HG-5 column (ϕ 20 × 250 mm, Nomura Chemical Co., Ltd., Aichi, Japan) with 60% methanol aq. (containing 0.01% AcOH) to afford **7** (1.1 mg, 59%) as a colorless oil (Supplementary Figure 25&26). ^{1}H NMR (700 MHz, CD_3OD, 3:1 mixture of oxime isomers, data for major isomer[2]) δ 6.46 (s, 1H), 3.81 (s, 3H), 2.92 (dt, J = 12.1, 6.1 Hz, 1H), 2.67–2.62 (m, 2H), 2.34 (dq, J = 11.2, 8.8 Hz, 1H), 2.28–2.12 (m, 2H), 1.76 (dt, J = 12.7, 4.6 Hz, 1H), 1.63 (quintet, J = 7.3, 2H), 1.55–1.37 (m, 6H), 1.17–1.12 (m, 2H), 1.02 (t, J = 7.3 Hz, 3H), 1.01 (t, J = 7.3 Hz, 3H). ^{13}C NMR (175 MHz, CD3OD) δ 175.2, 172.0, 169.7, 169.2, 138.3, 136.5, 61.7, 42.5, 40.7, 39.0, 38.9, 33.0, 31.0, 30.2, 29.2, 27.5, 23.3, 21.7, 13.8, 11.6. IR (film) cm^{-1}: 3315, 2961, 1700, 1656, 1624, 1517, 1459, 1277, 1182. HRMS (ESI, positive) *m/z* $[M+Na]^+$ calcd for $C_{19}H_{28}N_2O_4Na$ 371.1941, found 371.1939.

Compound **8**: *O*-phenylhydroxylamine hydrochloride (127 mg, 872 μmol) was added to a stirred solution of *ent***6** (27.8 mg, 87.0 μmol) in dry pyridine (1.0 mL). The mixture was stirred at r.t. for 2.5 h. Then, the mixture was diluted with distilled water and extracted with EtOAc. The combined organic layers were washed with distilled water and brine; dried over anhydrous Na_2SO_4; and concentrated *in vacuo*. The residue was purified by HPLC using a Develosil ODS-HG-5 column (ϕ 20 × 250 mm) with a linear gradient (methanol (0.01% AcOH):H_2O (0.01% AcOH) = 70:30 (10 min) to 85:15 (70 min), the ratio of oxime isomers was about 2:1, R_t = 49 min as major isomer, R_t = 46 min as minor isomer) to afford **8** (22.1 mg, 55% for major isomer; 5.6 mg, 14% for minor isomer) as a white solid (Supplementary Figure 27&28). The major isomer was used for the following *in vitro* and *in planta* assays. ^{1}H NMR (400 MHz, CD_3OD, data for major isomer) δ 7.28-7.25 (m, 2H), 7.13-7.25 (m, 2H), 6.96 (tt, J = 7.4, 1.1 Hz, 1H), 6.50 (s, 1H), 3.02 (dt, J = 11.8, 7.3 Hz, 1H), 2.93–2.83 (m, 2H), 2.58 (dq, J = 12.1, 8.9 Hz, 1H), 2.32 (dt, J = 12.1, 7.3 Hz, 1H), 2.24 (m, 1H), 1.87 (dt, J = 13.1, 4.6 Hz, 1H), 1.64 (quintet, J = 7.5 Hz, 2H), 1.57–1.42 (m, 5H), 1.26 (q, J = 13.1 Hz, 1H), 1.16 (dd, J = 9.2, 4.7 Hz, 1H), 1.03 (t, J =

7.5 Hz, 6H (CH_3×2)). ^{13}C NMR (175 MHz, CD_3OD) δ 175.0, 173.0, 172.0, 161.1, 138.5, 136.3, 130.2, 122.8, 115.5, 42.7, 39.3, 39.0, 38.9, 33.1, 31.0, 30.2, 29.2, 28.2, 23.4, 21.7, 13.8, 11.6. IR (film) cm^{-1}: 3306, 2963, 1698, 1656, 1624, 1593, 1211, 1158. HRMS (ESI, positive) *m/z* $[M+Na]^+$ calcd for $C_{24}H_{30}N_2O_4Na$ 433.2098, found 433.2098.

Compound **9**: *O*-benzylhydroxylamine hydrochloride (23.9 mg, 150.0 μmol) was added to a stirred solution of *ent***6** (1.5 mg, 4.7 μmol) in dry pyridine (0.5 mL). The mixture was stirred at r.t. for 13 h. Then, the mixture was diluted with distilled water and extracted with EtOAc. The combined organic layers were washed with distilled water and brine; dried over anhydrous Na_2SO_4; and concentrated *in vacuo*. The residue was purified by HPLC using a Develosil ODS-HG-5 column (ϕ 20 × 250 mm) with a linear gradient (methanol (0.01% AcOH):H_2O (0.01% AcOH) = 70:30 (10 min) to 85:15 (70 min), R_t = 40–42 min for the mixture of oxime isomers) to afford **9** (0.97 mg, 52%) as a white solid (Supplementary Figure 29&30). ^1H NMR (700 MHz, CD_3OD, 3:1 mixture of oxime isomers, data for major isomer) δ 7.35–7.32 (m, 4H), 7.29–7.26 (m, 1H), 6.46 (s, 1H), 5.05 (d, *J* = 12.5 Hz, 1H), 5.04 (d, *J* = 12.5 Hz, 1H), 2.92 (dt, *J* = 12.5, 6.4 Hz, 1H), 2.73–2.64 (m, 2H), 2.40 (dq, *J* = 11.4, 9.0 Hz, 1H), 2.28–2.10 (m, 2H), 1.74 (dt, *J* = 13.0, 4.6 Hz, 1H), 1.64 (quintet, *J* = 7.3 Hz, 2H), 1.54–1.28 (m, 7H), 1.16–1.11 (m, 2H), 1.02 (t, *J* = 7.3 Hz, 3H), 1.00 (t, *J* = 7.3 Hz, 3H). ^{13}C NMR (175 MHz, CD_3OD) δ 175.9, 172.3, 171.1, 170.5, 140.4, 139.2, 137.3, 130.1, 129.7, 129.5, 77.3, 43.3, 40.2, 39.8, 39.7, 33.8, 31.7, 31.0, 30.0, 28.7, 24.1, 22.5, 14.6, 12.4. IR (film) cm^{-1}: 3315, 2963, 1698, 1657, 1625, 1516, 1455, 1273, 1201. HRMS (ESI, positive) *m/z* $[M+Na]^+$ calcd for $C_{25}H_{32}N_2O_4Na$ 447.2254, found 447.2255.

Supplementary References

1) Okada, M.; Ito, S.; Matsubara, A.; Iwakura, I.; Egoshi, S.; Ueda, M. Total syntheses of coronatines by exo-selective Diels–Alder reaction and their biological activities on stomatal opening. *Org Biomol Chem* **7**, 3065-3073 (2009).

2) Monte, I. *et al.* Rational design of a ligand-based antagonist of jasmonate perception. *Nat Chem Biol* **10**, 671-6 (2014).
