# Supplementary material for: A rationally designed JAZ subtype-selective agonist of jasmonate perception
Source: Nat Commun. 2018 Sep 7;9:3654. doi: 10.1038/s41467-018-06135-y (PMC6128907; doi:10.1038/s41467-018-06135-y)
Supplement: Supplementary file 3 — Description of Additional Supplementary Files [file 41467_2018_6135_MOESM3_ESM.pdf]

Description of Additional Supplementary Files:

Supplementary Data 1: Microarray analysis. Microarray data was normalized by Gene Spring v14.9 through quantile normalization and log 2 transformation. Oneway ANOVA was performed and only genes with FDR values less than 0.5 were shown. Further Tuckey's HSD test with FDR was performed for individual combinations. The *p* value less than 0.05 are shown, while those with a value of 0.05 or greater are shown as blank.

Supplementary Data 2: Gene ontology enrichment analyses. These data were carried out using the PANTHER (protein annotation through evolutionary relationship) classification system database.
